# Further improvement in London’s air quality demands more than the Ultra Low Emission Zone policy

**DOI:** 10.1038/s44407-025-00030-9

**Published:** 2025-10-22

**Authors:** Chengxu Tong, Yuqing Dai, Matthew Cole, Robert J. R. Elliott, Suzanne E. Bartington, Bowen Liu, Zongbo Shi

**Affiliations:** 1 School of Geography, Earth and Environmental Science, University of Birmingham, Birmingham, UK; 2 Department of Economics, University of Birmingham, Birmingham, UK; 3 Department of Applied Health Research, University of Birmingham, Birmingham, UK; 4 Department of Management, University of Birmingham, Birmingham, UK

**Keywords:** Climate-change policy, Environmental economics, Environmental sciences, Environmental social sciences

## Abstract

Traffic emission is one of the most important sources of urban air pollution. Low emission zone (LEZ) is a flagship policy intervention to reduce urban emissions in many parts of the world. However, it is challenging to evaluate the effectiveness of such policies, with different methods resulting in several times in discrepancies. Here, we employed a causal framework to quantify the effects of Ultra-LEZ (ULEZ, 2019, central London) and its city-wide expansion (2023) on air quality in London. We found that the 2019 ULEZ led to 19.6% and 8.2% (~13.3 and 2.7 µg m^−3^) reductions in nitrogen dioxide (NO_2_) at traffic and urban background sites, respectively, in the three months after its implementation within central London, with positive spillover effects across Inner and Outer London. However, the ULEZ 2023 expansion showed no detectable impact on NO_2_. Furthermore, we found no significant benefits of ULEZ on fine particle (PM_2.5_) and PM_2.5_, and NO_2_ remained well above World Health Organization air quality guideline. Our results illustrate that ULEZ alone is insufficient to clean up the air in London and emissions from other sources such as domestic, commercial and industrial emissions and regional pollution should also be tackled.

## Introduction

Air pollution poses a significant global health risk, contributing to 4.2 million premature deaths worldwide with 99% of the world’s population living in areas that failed to meet the World Health Organisation’s (WHO) air quality guidelines in 2019^1,2^. The UK’s air quality has significantly improved in the last 20 years but there are still frequent non-compliances, particularly of nitrogen dioxide (NO_2_) and particulate matter (PM_2.5_), of air quality objectives in some urban areas. Therefore, a large variety of clean air actions have been implemented across the world to mitigate air pollution impacts.

In urban areas, NO_2_ and PM_2.5_ are among the most important air pollutants. NO_2_ has a relatively short atmospheric lifetime, particularly in the summer. This means that the effects of emission reductions are mostly local, especially in warmer seasons^3^. In contrast, PM_2.5_ can remain in the atmosphere for several days to a week and originates from a wider range of sources, including traffic, residential and commercial heating, as well as long-range transport^4^. Road traffic is often one of the major sources of nitrogen oxides (NO_X_, summation of nitrogen oxide NO and NO_2_) and NO_2_ emissions and is consequently a target for policy interventions. For example, Low Emission Zones (LEZs) are designed to reduce the number of non-compliant vehicles on the road and thus air pollutant emissions. Over the past decade, nearly 200 LEZs have been implemented across Europe, with mixed outcomes regarding air quality improvement^5^. For instance, cities like Munich (Germany) and Copenhagen (Denmark) have seen reductions in vehicular air pollution^6–8^, whereas Amsterdam (Netherlands) showed no significant effect^5,9^. In the UK, multiple cities were mandated to introduce LEZ schemes as a necessary measure to achieve statutory compliance with NO_2_ air quality objectives^10^. In London, the introduction of the LEZ in a phased approach from 2008 initially targeted heavy freight vehicles (and subsequently vans and minibuses), leading to statistically significant though relatively small improvements in air quality, including a 2.5–3.1% reduction in particulate matter, although no discernible reduction in NO_X_ was observed^11^. Beyond LEZ-type interventions targeting road transport, additional regulatory measures have also been introduced in London to address other major sources of PM_2.5_. These include enhanced controls on wood burning and other significant combustion sources through strengthened smoke control zones and proposed city-level emission standards^12^.

**Fig. 1:**
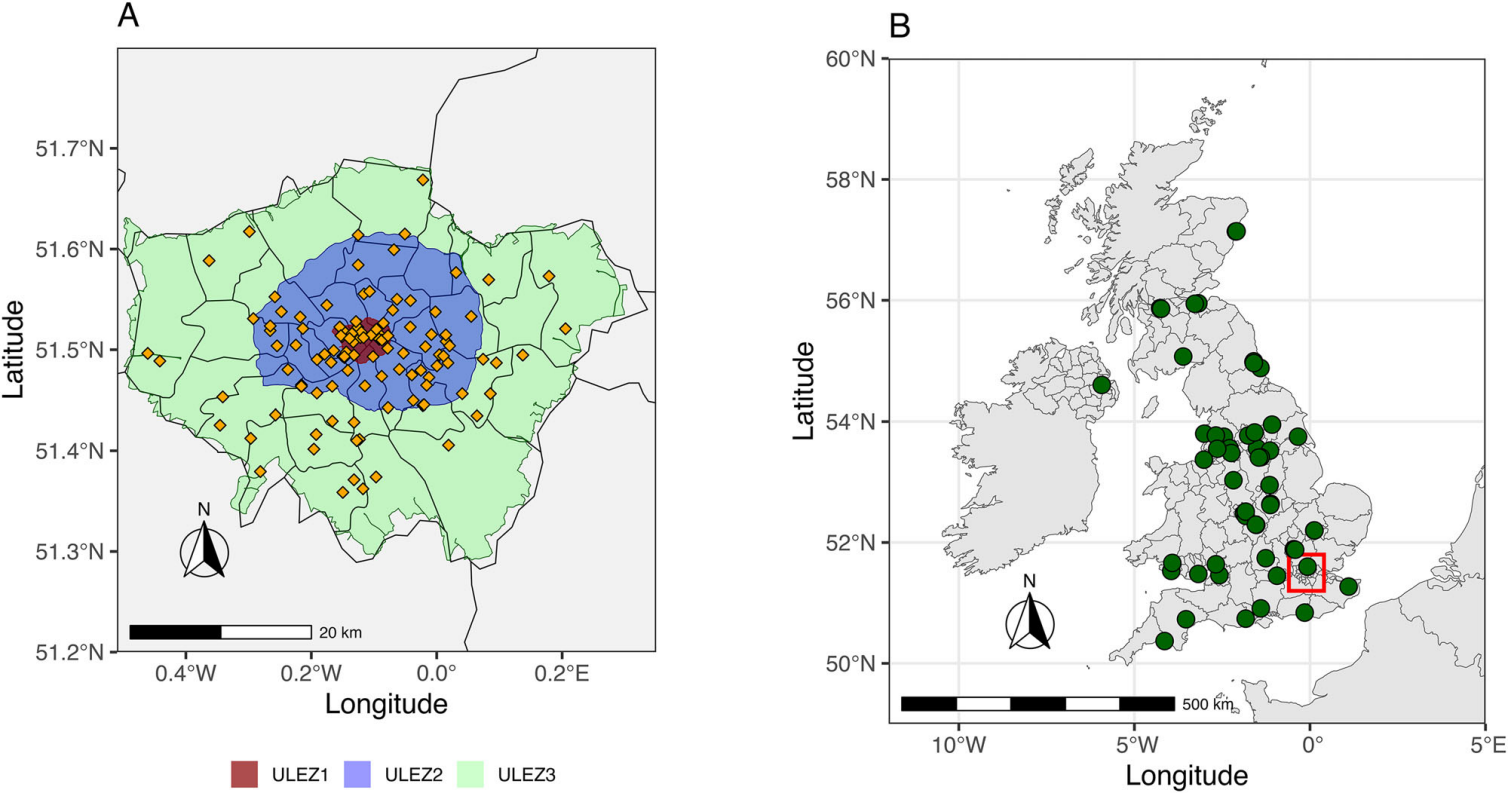
Maps showing the monitoring sites used for this study. **A** Map of central, inner and outer London covered by ULEZ1 and the ULEZ expansions (ULEZ2 and ULEZ3). **B** Monitoring sites across the UK used as controls.

Building on this and the previous Congestion Charge zone (launched in 2003) in central London (see Fig. 1A for the area coverage), the Mayor of London implemented the Ultra Low Emission Zone (ULEZ), referred to as ULEZ1, in April 2019, initially covering central London (see Fig. 1A). This policy imposed stricter regulations and applied to most vehicle types, including motorcycles, cars, vans, and minibuses. Specifically, it required Euro 3 for motorcycles and mopeds, Euro 4 for petrol cars and vans, and Euro 6 for diesel cars and vans. Drivers of vehicles that did not meet these standards were required to pay a £12.50 daily charge (in addition to the existing Congestion Charge). Prior to the implementation of ULEZ1, around 39% of vehicles (~35,578) entering central London were non-compliant with the emissions standards^13^. Note that lorries and specialist heavy vehicles (over 3.5 tonnes gross vehicle weight, GVW), buses, minibuses, and coaches, were regulated under the separate London Low Emission Zone (LEZ) scheme, which was introduced in 2008 and was later strengthened in March 2021. The ULEZ was expanded in October 2021 to cover inner London (ULEZ2) and again in August 2023 to include the entire Greater London area (ULEZ3, Fig. 1A), making it one of the largest ULEZ schemes globally in terms of both geographical coverage and the number of vehicles affected^14^. Given the scale and significance of the ULEZ, and with many other UK city administrations actively either implementing or considering similar policies, a rigorous evaluation of the ULEZ scheme’s effectiveness is crucial. Despite its prominence, the impact of the ULEZ remains a subject of debate. Existing studies have used a variety of different methods and reported a wide range of air quality outcomes^13,15,16^, though the vast majority of these studies do not use causal inference to identify the effects of ULEZ.

Identifying the causal effects of ULEZ is challenging for two reasons. First, confounding factors such as meteorological conditions can often mask any policy impact. Second, as air pollution levels have been generally decreasing across the UK in recent years^17^ associated with clean air actions including the transition to cleaner vehicles, a simple before-and-after comparison of ULEZ implementation cannot provide accurate results. The lack of a clear counterfactual complicates efforts to determine what pollution levels would have been in the absence of ULEZ measures. To address these challenges, machine learning-based weather normalisations (so-called ‘deweathering’) were applied to remove the influence of meteorological conditions to uncover the underlying pollution trends related to emissions only^18^. The machine learning model incorporated both meteorological parameters and time-related variables as emission proxies to capture pollutant concentrations under different emission scenarios and meteorological conditions. All air pollutants were modelled separately using data from the same policy evaluation window, ensuring consistency in temporal comparison. This method has been used to evaluate the impact of COVID-19 lockdown on air quality globally^19–21^. Vu et al.^22^ provided an updated weather normalization approach by selectively replacing meteorological factors over specific time periods, which is applied in this research. However, while the ‘deweathering’ helps decouple meteorological influences, it cannot address other confounding factors such as socioeconomic variables, nor can it establish a robust causal link between policy interventions and outcomes. For this reason, more rigorous evaluation techniques are needed. Ben-Michael et al.^23^ proposed an augmented synthetic control method (ASCM), which offers a more robust framework by constructing counterfactual scenarios. These scenarios allow for more accurate estimation of policy impacts by comparing policy implementation areas to control groups, where the policy was not implemented. Combining the “deweathering” technique with ASCM thus offers the potential to identify causal policy effects on air pollution patterns^24^.

This study uses air quality data from 124 sites across London (Fig. 1A and Supplementary Table S1) to assess the direct impacts of the initial implementation of ULEZ in 2019 (ULEZ1), and the ULEZ second expansion in 2023 (denoted as “ULEZ3”) on key air pollutants, including NO_2_, NO_X_ and fine particles (PM_2.5_). We exclude the ULEZ first expansion in 2021 (denoted as “ULEZ2”) as this study period overlaps with COVID-19 lockdown. Additionally, air quality data from 60 other UK sites (Fig. 1B and Supplementary Table S2) are collected to construct the counterfactual scenario using ASCM, representing pollution levels in London in the absence of ULEZ. This approach allows us to assess not only the direct effects of the ULEZ but also potential spillover effects occurring in areas outside the ULEZ, as proposed in previous studies^25,26^.

## Results

### Ultra low emission zone effects on air pollutants

**Fig. 2:**
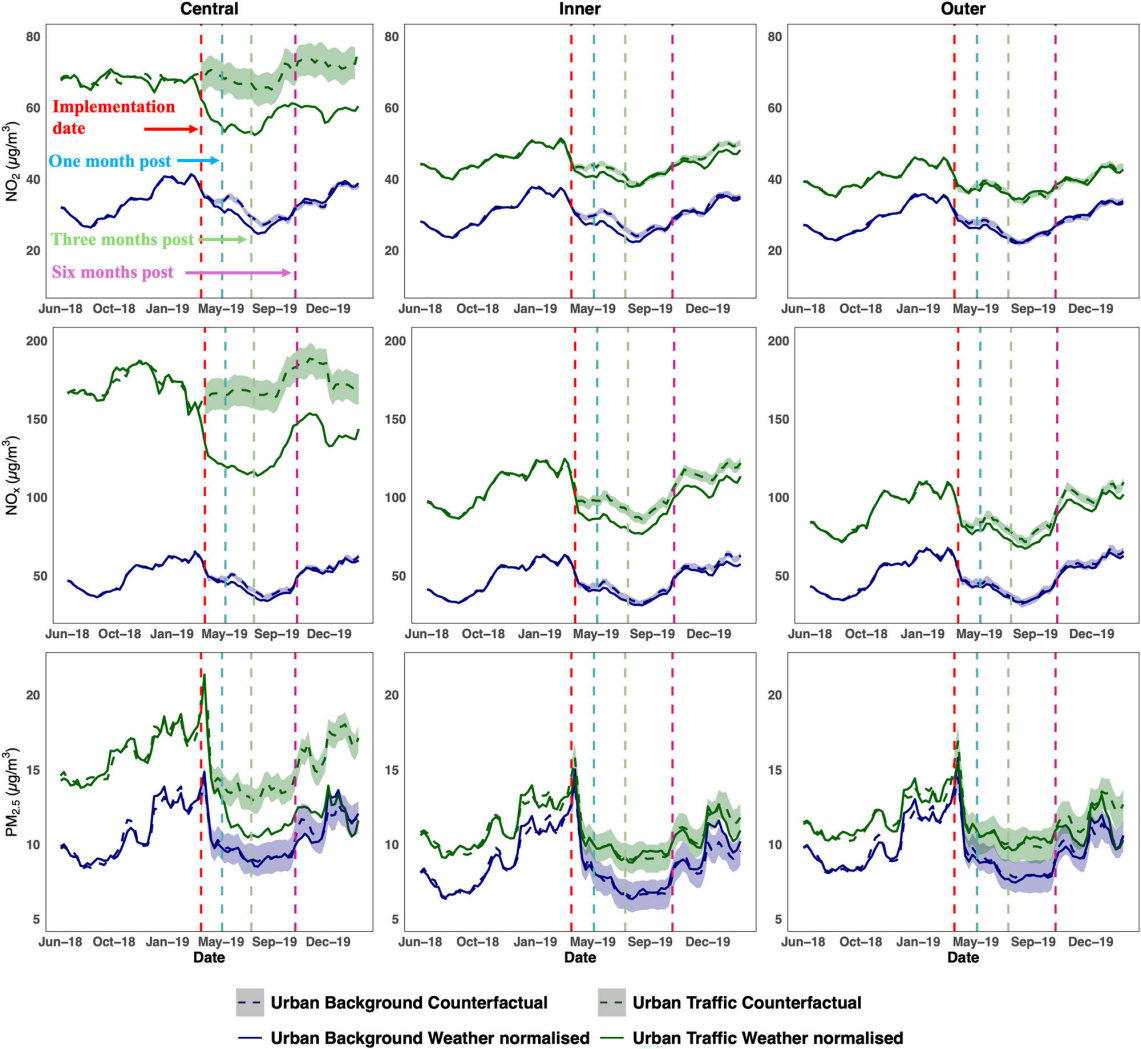
Weather normalised and counterfactual concentrations of NO_2_, NO_X_ and PM_2.5_ at urban background and traffic sites in central, inner and outer London (see Fig. 1A for a map), respectively, following ULEZ1 implementation. The red dotted vertical line represents the date of ULEZ1 implementation (8^th^ April 2019) and the dashed lines represent one month, three months and six months after policy implementation. The blue (urban background) and green (urban traffic) trends represent the weather normalised pollution levels and their counterfactual levels, respectively. The shaded blue and green areas represent the 95% point-wise confidence intervals.

Figure 2 illustrates the weekly averaged weather-normalised and counterfactual concentrations of NO_2_, NO_X_ and PM_2.5_ across urban background (UB) and traffic (UT) sites within central, inner, and outer London, following the implementation of ULEZ1 in April 2019. The analysis spans from June 2018 to March 2020, including more than ten months of pre-intervention data to establish a baseline for the counterfactual trend. The difference in concentration between weather normalised and synthetic counterfactual trend after the intervention (i.e., ULEZ1 and 3) represents the real impact of policies^27,28^. We calculated both the average absolute effect (“Average Treatment Effect” of ULEZ) and relative effect (percentage changes) over one, three, six, nine, and 11 months, with 11 months being the longest timeframe available for analysis before the COVID-19 lockdown in 2020. These results are detailed in Supplementary Tables S3 and S4.

In Central London, the weather-normalised concentration of NO_2_ exhibited a clear divergence from the counterfactual concentration following ULEZ1 implementation (Fig. 2). A similar trend is observed at the urban background sites, though less pronounced. Specifically, NO_2_ at traffic sites within the central London decreased by 19.6% (~13.3 µg m^−3^) three months after the initial ULEZ1 implementation and 17.8% (~12.4 µg m^−3^) after 11 months. Inner and outer London experienced smaller declines in NO_2_, with inner London experiencing a 5.4% reduction (~2.3 µg m^−3^) after three months and 3.3% (~1.4 µg m^−3^) after 11 months. In contrast, the ULEZ1 had smaller effects on air quality at urban background sites. NO_2_ concentrations decreased by 8.2% (~2.7 µg m^−3^) in central London, 8.6% (~2.5 µg m^−3^) and 4.6% (~1.3 µg m^−3^) in inner London and outer London after three months (Supplementary Tables S3, S4).

NO_X_ concentrations followed similar trends to NO_2_ after ULEZ1 implementation. NO_X_ in Central London traffic sites decreased by 28.8% (~48.1 µg m^−3^) after three months and 24.2% (~41.7 µg m^−3^) after 11 months (see Fig. 2 and Supplementary Tables S3, S4). Reductions were also observed in inner and outer London, though to a lesser extent. In inner London, ULEZ1 NO_X_ decreased by 12.2% (~11.8 µg m^−3^) after three months and 9.5% (~9.8 µg m^−3^) after 11 months. In outer London, NO_X_ fell by 6.3% (~5.2 µg m^−3^) after three months and 6.1% (~5.5 µg m^−3^) after 11 months. Urban background sites experienced smaller reductions, with central London showing a 6.6% decline (~3.1 µg m^−3^) after three months and 3.9% (~1.9 µg m^−3^) after 11 months. In inner London, NO_X_ levels decreased by 6.5% (~2.7 µg m^−3^) and 5.3% (~2.5 µg m^−3^) after three months and 11 months of ULEZ1. For both NO_2_ and NO_X_, the most substantial effects were typically observed within the first three months following the policy’s introduction, regardless of the region.

In contrast to NO_2_ and NO_X_, the impact of ULEZ1 on PM_2.5_ was limited, with only traffic sites in central London showing a 12.7% reduction (~1.8 µg m^−3^) in PM_2.5_ after three months (Fig. 2). No statistically significant reductions in PM_2.5_ were detected at urban background sites or in inner and outer London.

**Fig. 3:**
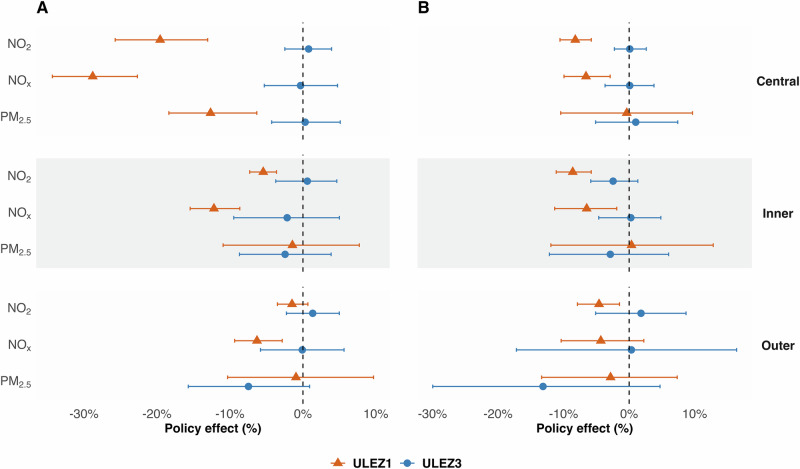
Policy effect of ULEZ1 and ULEZ3 after three months in London. Estimated effects at (**A**) Urban Traffic and (**B**) Urban Background sites across central, inner and outer London. Significant effects on NO_2_ and NOx were detected following ULEZ1 implementation but not ULEZ3 at both central and inner London. Except at traffic site following ULEZ1 implementation, no statistically significant effect was detected for PM_2.5_ following either intervention at either urban background or traffic sites.

Focusing now on the impact of the second expansion (ULEZ3), Fig. 3 compares the relative changes at urban background and traffic sites for three months following the ULEZ1 and ULEZ3 implementation to quantify (average) relative causal effect. Unlike ULEZ1, concentrations of NO_2_, NO_X_, and PM_2.5_ did not show any significant changes comparing to counterfactuals following subsequent expansions (ULEZ3). Detailed data for absolute and relative changes are provided in Supplementary Tables S5, S6. Additionally, as shown in Supplementary Fig. S7, no significant difference was observed between the counterfactual trend and the weather-normalised trend following ULEZ3 implementation. This pattern is evident across both urban background and urban traffic sites for NO_2_, NO_X_, and PM_2.5_.

## Discussion

This study evaluates the effects of both the London ULEZ and its subsequent expansion using a causal inference framework. We found that the implementation of the ULEZ1 led to significant reductions in NO_2_ and NO_X_ levels within central London, with decreases of 19.6% and 28.8%, respectively, at traffic sites during the first three months following the policy implementation. However, the second ULEZ expansion in 2023 (ULEZ3) did not result in statistically significant changes on NO_2_ or NO_X_ levels. Additionally, we found no statistically significant impact of ULEZ1 on NO_2_ and NO_x_ and the impact of its expansion (ULEZ3) on PM_2.5_ was minimal (except at the traffic site after ULEZ1).

Supplementary Table S7 compares our results with previous studies conducted over the same period and summarizes the methods used. The Greater London Authority^29^, employing a Difference-in-Difference (DID) method, reported a 28 µg m^−3^ (34.0%) reduction in NO_2_ levels in central London within three months following ULEZ1 implementation, compared to a no-ULEZ scenario. These reductions are larger than our findings over the same period, which show a ULEZ1 effect of 11.4 µg m^−3^ (17.0%) at urban traffic sites. This discrepancy may be due to the Greater London Authority’s choice of 2017 as the baseline, which captured the combined effects of both the Toxicity Charge (T-Charge)—targeting high-emission vehicles that do not meet Euro 4 standards—and ULEZ1^15^. Note selecting outer London sites as the control group may introduce bias, as these sites might also be influenced by ULEZ1. We also noted that their analysis relied on smoothing techniques and the assumption that the control and treatment groups were equally affected by meteorological conditions, yet lacked a systematic adjustment for meteorological influences on air pollutant concentrations, thereby limiting its ability to robustly decouple policy effects from variations driven by weather. Similar to Greater London Authority^29^, Prieto-Rodriguez et al.^16^ applied the DID method, incorporating meteorological factors as control variables in their regression analysis, and reported a 19.0% reduction in NO_2_ levels in urban traffic sites until 29^th^ February 2020, compared to 17.3% in our analysis. The difference was pronounced for the inner and outer London, with Prieto-Rodriguez et al.’s finding of a 15.5% reduction, whereas our analysis showed only 3.2%. The higher values they obtained are likely due to challenges in meeting pre-parallel trends and/or the limited flexibility in controlling for meteorological conditions within the DID framework, particularly when compared to machine learning-based approaches. Combining meteorological normalization and the Regression Discontinuity Design (RDD) method, Ma et al.^15^ found a 1.6% decline in NO_2_ for urban traffic sites (until 31^st^ January 2020) (i.e., smaller than the 17.4% reduction in our study) and reported a 3.0% reduction in PM_10_ levels. While RDD is effective for estimating sharp and localized effects at policy cut-offs, the ASCM offers dynamic counterfactuals across multiple units within a causal framework for assessing long-term policy impacts^23,30^. After applying weather normalisations, ASCM also generated synthetic counterfactuals from non-London sites, facilitating robust causal inference regarding ULEZ’s effects.

Following ULEZ1’s implementation, concentrations of both NO_X_ and NO_2_ decreased in central London, with a more pronounced reduction in NO_X_ levels. NO_X_ consists of nitric oxide (NO) and nitrogen dioxide (NO_2_), and vehicle NOx emissions are predominantly in the form of NO, which is readily oxidized to NO_2_ in the atmosphere in the presence of ozone. Consequently, assessing reductions in NO_2_ alone involves NO-to-NO_2_ transformation. Therefore, NO_X_ is considered a more reliable indicator of traffic emission changes^31^. The substantial reduction in NO_X_ across all areas of London, indicating that the policy had a sustained impact on NO_X_ levels. To better understand these changes in NO_X_ concentrations, this study analysed traffic increments calculated as the difference between urban traffic sites and urban background sites, which capture variations in the contribution of traffic emissions to NO_X_ levels and serve as a metric for assessing traffic-related pollution^32^. In our analysis, weather-normalised traffic increments for NO_X_ were compared between the pre-intervention period and the three months following the implementation of ULEZ, with the pre-intervention period consistent in duration with the causal model fitting. Substantial reductions of 48.2 µg m^−3^ (39.1%) and 12.4 µg m^−3^ (21.4%) were observed in central and inner London, respectively (Supplementary Fig. S8), agreeing with our primary findings, which are 48.1 µg m^−3^ and 11.8 µg m^−3^ separately (Supplementary Tables S3, S4). In contrast, no statistically significant changes were observed following ULEZ3 (Supplementary Fig. S9). The significant decreases in traffic increments following ULEZ1 support the conclusion that these reductions are a direct effect of the policy, further demonstrating its efficacy.

**Fig. 4:**
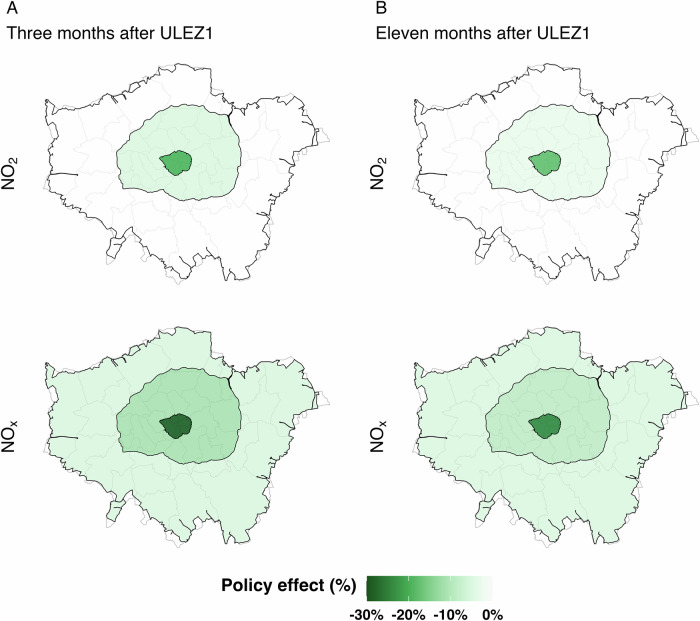
ULEZ1 implementation and spillover effects on NO_2_ and NO_x_ in London. Estimated effects are shown for (**A)** three months and (**B**) eleven months after ULEZ1 implementation at urban traffic sites, in central, inner and outer London areas for NO_2_ and NO_X_ indicating the "positive" ULEZ1 effect across central to outer London.

Although ULEZ1 caused significant reductions in NO_X_ concentrations, the expansion of ULEZ (ULEZ3) had no statistically detectable impact. This differences between ULEZ1 and ULEZ3 are likely associated with spillover effects, traffic compliance rates, and the sources of NO_X_ emissions. Our research demonstrates that ULEZ1 had a significant impact not only within the designated traffic restriction zone but also on areas of London beyond the policy boundary. Figure 4 presents the average policy effect on NO_2_ and NO_X_ levels at urban traffic monitoring sites across central, inner, and outer London. The spatial pattern suggests a diminishing positive spillover effect as distance from central London increases (Fig. 4). Significant reductions in NO_2_ were observed in central and inner London. This smaller reduction in NO_2_ relative to NO_X_ likely reflects the influence of atmospheric chemistry. After emission, NO is oxidised in the atmosphere, primarily through its reaction with O_3_ to form NO_2_^31^. This chemical conversion can mask part of the policy’s impact on atmospheric NO_2_, especially in areas with smaller absolute reductions such as outer London, where the changes were not statistically significant. In contrast, NO_X_ showed more substantial and consistent declines across all regions, remaining statistically significant even 11 months after the ULEZ1 implementation (Fig. 4), highlighting ULEZ1’s impact on vehicular emissions extended beyond central London. This suggests that residents outside the initial ULEZ boundary had already modified their transportation behaviours, such as adopting compliant vehicles, leaving less room for reducing traffic NO_X_ emissions in the target areas (inner and outer London) when ULEZ expansions were introduced.

After the introduction of ULEZ1, there was a substantial decrease in non-compliant vehicles operating in central London (Supplementary Fig. S10 and Supplementary Table S8). The average proportion of non-compliant vehicles fell from 39.1% in March 2019 to 29.3% in April 2019, and further decreased to 25.8% by September 2019^13^. As more vehicles complied with higher emission standards, vehicle-related NO_X_ emissions decreased^33^, explaining the efficacy of ULEZ1 policy. The proportion of non-compliant vehicles on the road decreased substantially over time, from 23.1% in January 2020 (Supplementary Table S8) to 7.4% by the implementation of ULEZ3 (Supplementary Table S10). Several factors could have contributed to this trend. For example, to encourage residents to replace non-compliant vehicles, the London government has offered a substantial scrappage scheme^34^ and demonstrated strong commitment to expanding the ULEZ, which could have impacted replacement of non-compliant vehicles before the implementation of ULEZ 3 (Supplementary Tables S9, S10). Additionally, ULEZ3 was implemented in the post-pandemic period, during which commuting patterns had already been reshaped by the COVID-19 lockdown. Research in the UK shows that some commuters who previously relied on cars or public transportation began considering a shift to active transportation modes, such as walking or cycling^35^. Following the implementation of ULEZ3, the number of non-compliant vehicles only fell by 2.7–4.7% by September 2023 (Supplementary Table S10). Although this is expected to lead to reduced NO_X_ emissions, such an impact is not detectable when considering the counterfactuals (Fig. 3).

To better understand the broader sources of NO_X_ emission in London, we examined data from the London Atmospheric Emission Inventory^36^, presented in Supplementary Fig. S11. In 2013, road transport contributed 1046.1 tonnes (37.6%) of NO_X_ emissions in central London, compared to 1550.0 tonnes (55.7%) from industrial, commercial, and domestic sources. In inner and outer London, road transport contributed 10,349.6 tonnes (60.8%) and 14,751.7 tonnes (53.5%), respectively. By 2019, on-road NO_X_ emissions in central, inner, and outer London had decreased by 52.7%, 50.9%, and 32.8%, down to 494.8, 5086.0, and 9911.5 tonnes, respectively. Correspondingly, the share of on-road NO_X_ emissions relative to total emissions in these regions declined by 14.1%, 15.9%, and 9.0%, respectively. In contrast, the proportion of emissions from industrial, commercial, and domestic sources increased by 10.7%, 13.9%, and 4.9%^37^. These data indicate that, although ULEZ has reduced traffic-related NO_X_ emissions (Supplementary Fig. S11), the substantial contribution of non-traffic sources has limited the overall reduction in ambient NO_X_ concentrations. This is qualitatively consistent with our results that despite ULEZ achieving a reduction in the number of high-emission vehicles, further decreases in NO_X_ levels were limited by expanding the ULEZ to inner and outer London.

Our finding reveals that ULEZ1 led to a reduction in PM_2.5_ levels at traffic sites, demonstrating the policy’s efficacy in reducing primary PM_2.5_ emissions from road transport by limiting the number of non-compliant vehicles in the area. Consistent with these findings, Ma et al.^15^ observed particulate matter reductions at selected sites after the policy implementation. However, no detectable changes were observed at urban background sites in central London, indicating that, unlike NO_2_ and NO_X_, PM_2.5_ reductions from urban traffic sites have limited impacts at the urban background. Supplementary Fig. S12 illustrates that road transport accounts for only 11% of PM_2.5_ sources in central London, while industrial and commercial activities account for 82%. A significant proportion of traffic-related emissions originates from non-exhaust sources, including brake wear, tyre wear, and road surface abrasion, which currently account for ~60% and 73% (by mass) of primary PM_2.5_ and PM_10_ emissions, respectively, from road transport in the UK^38–40^. Furthermore, modelling studies have shown that long-range transported PM_2.5_ from continental Europe contributes ~28% of total PM_2.5_ concentrations in London, representing a substantial component of the region’s secondary particulate pollution^41^. These factors explain why modest PM_2.5_ reductions from road transport have limited impact on urban background concentrations.

Our results demonstrate the effectiveness of ULEZ policies in reducing vehicle emissions, particularly NO_2_ and NO_X_. ULEZ encourages the transition from a minority of high-polluting vehicles to cleaner alternatives. This likely generated broad societal benefits, including improvement in both physical and mental health, increased productivity, improved ecosystem services, natural capital, biodiversity, and reduced crime rates and altered tourism preferences^42^.

It is important to note that this study did not assess the potential effects of ULEZ2 or the strengthened LEZ regulation targeting heavy goods vehicles (HGVs), as these policies fell outside the specific evaluation windows used in our analysis due to the impacts of COVID lockdowns. While these interventions may have contributed to further reductions in NO_2_ and PM_2.5_ concentrations, our findings show that following ULEZ3 (Supplementary Table S6), NO_2_ levels in central, inner, and outer London remain substantially above the WHO Global Air Quality health-based guidelines^1^ of 10 µg m^−3^, ranging from 13.9 µg m^−3^ to 25.4 µg m^−3^ at urban background sites and 15.7 µg m^−3^ to 44.8 µg m^−3^ at urban traffic sites in 2023 following the introduction of ULEZ3. Furthermore, despite the implementation of ULEZ1 and ULEZ3, PM_2.5_ levels remained high in 2023, with urban background sites ranging from 6.8 µg m^−3^ to 9.8 µg m^−3^ and urban traffic sites from 7.4 µg m^−3^ to 11.6 µg m^−3^ across greater London–above the WHO’s recommended annual mean of 5 µg m^−3^. The challenge lies in addressing PM_2.5_’s diverse sources, which extend beyond transport to include regional pollution, agriculture, industries, and domestic woodburning^39,41,43^. Even as electric vehicles become more prevalent, non-exhaust emissions from tyre and brake wear, as well as road dust, will continue to contribute substantially to PM_2.5_ pollution^39,44^. Electrification of transport alone is therefore insufficient for a significant reduction in PM_2.5_ concentrations^44,45^.

Achieving further reductions in NO_2_ and PM_2.5_ will require strengthening existing transport emission controls. This could involve upgrading both petrol and diesel vehicle requirements to the most stringent Euro 6e standard and potentially adopting Euro 7 in the future as it becomes available. In addition, city-wide regulatory approaches to reduce vehicle idling warrant further investigation to assess their potential impact on air quality, particularly in areas with high traffic density. However, tackling emissions from sectors beyond transportation is becoming increasingly more important. For instance, reducing vehicular mileages in urban areas and encouraging a shift to active transportation modes, such as walking and cycling, can more effectively reduce non-exhaust emissions, which is particularly crucial for lowering PM_2.5_ levels^44^. In addition, such policies will be essential for reducing CO_2_ emissions from the transport sector, which at present is a large contributor to carbon emissions with limited progress in recent years^46^. Other significant NO_X_ and PM_2.5_ sources, including industrial processes and residential woodburning should also be targeted to improve urban air quality^43,45^. Additionally, establishing regionally coordinated PM_2.5_ control standards with continental Europe could potentially mitigate the effects of cross-border transmissions^4^. Only by addressing emissions across multiple sectors can cities like London achieve their air quality targets and safeguard public health.

## Methods

### Air quality and meteorological data

In this study, monitoring sites in London were selected as the primary focus. The air quality data were sourced from two monitoring networks: the Automatic Urban and Rural Network (AURN) and the London Air Quality Network (LAQN). These networks provide open-source data that can be accessed through R package ‘Openair’^47^. A total of 124 sites in London were selected for analysis. In addition to the London sites, a total of 60 sites across other UK cities were also obtained from AURN and regional air quality networks sites. The location of the sites and the area covered by ULEZ are shown in Fig. 1. The details of each site are shown in Supplementary Tables S1 and S2. It’s important to note that not all sites have complete data for all the required pollutants, and data quality varies across sites. Consequently, we implemented rigorous data quality control measures^15^. For example, a day was considered valid only if more than 75% of hourly data were available for that day. Similarly, an air pollution monitoring station was included in the study only if over 80% of the days within the study period met the validity criteria.

Data were collected from both urban background sites and urban traffic sites across three zones, the initial implementation area, the inner area of London, and outer London. Aggregated data were then calculated for each region and site type, providing a comprehensive basis for assessing the overall impact of the Ultra Low Emission Zone (ULEZ) across Greater London. This approach allowed for an in-depth analysis of the ULEZ effect on urban background and urban traffic monitoring sites, as well as on multiple pollutants, including NO_2_, NO_X_, PM_2.5_.

Considering the timing of the ULEZ implementation and its two expansions, hourly data ranging from 8^th^ March 2018 to 8^th^ March 2020 were selected to analyse ULEZ1 implementation. For ULEZ3, data from 19^th^ July 2021 to 30^th^ April 2024 were used to evaluate its impacts. The first ULEZ expansion, implemented on October 25^th^, 2021 (ULEZ2), was excluded from this study, as its start date was close to the end of the COVID-19 lockdown on July 19^th^, 2021. This closing timing limited the availability of sufficient pre-policy data, restricting the ability to isolate and accurately assess ULEZ2’s effects within our study scope. The broader time range selected provides flexibility in assessing the policy’s impacts while reducing the potential confounding effects of the COVID-19 lockdown^27^. However, this timeframe design prevented the evaluation of the potential effects of ULEZ2 and the strengthened LEZ regulation targeting HGVs in March 2021.

Hourly meteorological data for the study were obtained from two sources: NOAA (National Oceanic and Atmospheric Administration) and ERA5 (European Centre for Medium-Range Weather Forecasts). NOAA Integrated Surface Database (ISD) provided observed data on temperature, relative humidity, wind speed, and wind direction from the nearest meteorological observation site for each city. ERA5 data were derived from the grid (0.25° × 0.25°), covering variables such as surface net solar radiation, total precipitation, boundary layer height, total cloud cover, and surface pressure. These meteorological parameters were chosen to offer comprehensive insights into energy balance, atmospheric moisture, stability, cloud cover, and pressure conditions, enhancing the model’s robustness within a machine learning framework^20,48^.

### Weather normalization approach

The random forest based meteorological normalization technique provides a flexible framework for accounting for the impact of weather on pollution levels. This modelling approach was initially introduced by Grange and Carslaw^18^ and further developed by Shi et al.^20^ to analyse the impact of Covid-19 lockdown policies. It has been demonstrated to be highly effective in evaluating the effects of short-term policies.

Following Shi et al.^20^, this study employs the RF based weather normalisation model to decouple the impact of meteorology on observed pollution levels. For each pollutant monitored at each site, an RF model was constructed to predict hourly pollution levels using the previously mentioned meteorological variables and time variables. Time variables were incorporated into the explanatory variables as proxies for time-related factors (e.g., emission intensity). We used Unix time as a linear trend component, Gregorian date (day of the year) as a seasonal component, day of the week as a weekly component, and hour of the day as a diel-cycle component, covering the period between 8^th^ March 2018 to 8^th^ March 2020 and 19^th^ July 2021 to 30^th^ April 2024. The hyperparameters for the RF model are consistent with previous studies^20,24^: a forest of 300 trees (n_tree = 300) and a minimum terminal node size of 5 (min_node_size = 5). We used 70% of the original dataset for training and the remaining 30% for testing. The RF models for all studied groups demonstrated low bias (close to 0) and high correlation coefficients (>0.7). The weather normalisation process was achieved by randomly replacing meteorological variables. For example, each trained RF predictive model (for each pollutant in each site) was used to predict hourly PM_2.5_ concentration 150 times, for every prediction, only the meteorological variables were randomly sampled without replacement. We adopted the sampling technique proposed by Vu et al.^22^, for instance, for 11:00 am on April 1, 2019, only meteorological variables from 11:00 am in the two weeks before and two weeks after this date for each year were selected. The 150 predictions for 11:00 am on April 1, 2019, were then aggregated using the arithmetic mean, and representing the pollution level under “average” meteorological conditions, i.e., the “weather normalised” pollution level for that hour. This weather normalization technique effectively normalizes the weather conditions while preserving seasonal and diurnal variations, enabling us to employ subsequent causal inference methods to evaluate the effects of ULEZ (Supplementary Figs. S1–S6).

### Augmented synthetic control method

Based on the quasi-experimental design idea presented in Cole et al.^49^ and Song et al.^48^, all the hourly weather normalised (WN) pollution levels are averaged into weekly frequency and then fed into the Augmented Synthetic Control Method (ASCM) to evaluate the causal effect of the policy. The concept of Synthetic Control Method (SCM) is to select a set of control groups that are not affected by the policy of interest to construct a counterfactual trend for the treatment group. Air quality sites in other major UK cities (outside London) were selected as the control group. These locations were carefully screened to ensure that no similar low-emission interventions or major air quality policies were implemented during the analysis period, thereby satisfying the “no interference” assumption required for ASCM^27,48^. These sites, located in other major UK cities, serve as suitable candidates for constructing London’s counterfactual trends, as they exhibit broadly similar traffic patterns and vehicle fleet compositions to London, yet are unaffected by the ULEZ intervention. In addition, sites showing abnormal pollution trends were removed to minimise estimation uncertainty and ensure model stability. These selection criteria ensured a satisfactory pre-policy fit of the model (Supplementary Table S2 shows the list of all the sites used in this study). The ‘true (causal)’ policy effect can then be evaluated by comparing the difference between the actual observed trend and the counterfactual trend after the policy implementation^30^. Separate counterfactual trends were constructed for ULEZ1 and ULEZ3 for central, inner and outer London, using ASCM models independently fitted to each policy period.

Following Ben-Michael et al.^23^ the Ridge ASCM approach is applied in this study. The Ridge ASCM enhances the pre-policy fitting of the counterfactual, thereby improving the accuracy of the estimation. Confidence levels are calculated using a Jackknife+ procedure, which sequentially excludes individual data points to evaluate the variability and uncertainty of the estimated values. The resulting confidence intervals provide a probabilistic boundary within which the true values are projected to reside, at a 95% confidence level. We use the following model to estimate the counterfactual WN concentrations at time *T*, ${\hat{Y}}_{1T}$:

1 $${\hat{Y}}_{1T}=\sum {\hat{w}}_{j}^{{scm}}{Y}_{{jT}}+\left({X}_{1}-\sum {\hat{w}}_{j}^{{scm}}{X}_{j}\right)\cdot {\hat{{\rm{\eta }}}}^{{\rm{Ridge}}}=\sum {\hat{w}}_{j}^{{aug}}{Y}_{{jT}}$$

where, 1 denotes the notation for treatment city and j denotes the control cities, the term ${Y}_{{jT}}$ signifies the air pollutant concentrations in the control group normalised for meteorological conditions at time *T*. ${X}_{1}$ and ${X}_{j}$ represent the vectors of pre-policy results for the treatment group and control group. ${\hat{{\rm{\eta }}}}^{{\rm{Ridge}}}$ are the coefficients derived from a ridge regression. ${\hat{w}}_{j}^{{scm}}$ denotes the weights estimated using the original Synthetic Control Method (SCM), ${\hat{w}}_{j}^{{aug}}$ indicates the estimated components of the augmented weights vector ${w}^{{aug}}$.

The causal effect ${Y}_{{ULEZ},T}$ caused by ULEZ at time *T* is:

2 $${Y}_{{ULEZ},T}={Y}_{1T}-{\hat{Y}}_{1T}$$

where, ${Y}_{1T}$ indicates the WN concentrations and ${\hat{Y}}_{1T}$ represents the counterfactual WN concentrations at time *T*.

By utilizing ASCM, the challenges associated with control group selection in traditional comparative experiments are effectively addressed. In this study, other sites across the UK that were not impacted by the ULEZ policy were selected as the control group. Separate counterfactuals were fitted for different site types, specifically urban traffic and urban background sites.

### Policy effect evaluation

In order to investigate the extent of air pollution reduction following the policy implementation, we averaged the policy effects across different time periods. The percentage change (P) used in this study is calculated as follows:

3 $$P=\frac{{C}_{{\bf{wn}}}-{C}_{{\bf{Counterfactual}}}}{{C}_{{\bf{Counterfactual}}}}\times 100 \%$$

where ${C}_{{\bf{wn}}}$ is the weather-normalization data averaged by the selected period, ${C}_{{\bf{Counterfactual}}}$ is the corresponding counterfactual concentrations calculated using different control groups.

This study excluded the first and second weeks following the policy implementation, when calculating the average effect. These initial weeks were considered as a transition period, during which changes could be substantial. Including this timeframe in the calculation might lead to either an overestimation or underestimation of the average policy effect^20^.

To scrutinize the anticipatory effect of ULEZ1, we analysed data from June 8^th^, 2018—the date corresponding to the announcements of both ULEZ1 implementation and its expansion^50^. Supplementary Fig. S13 presents findings indicating the absence of a significant announcement effect (anticipatory effect). This result can also be interpreted as a placebo test conducted over time, further reinforcing the robustness of the ULEZ1 results due to the lack of significant effects at other time points. In addition, we conducted a sensitivity analysis using 6–9 a.m. peak-hour data and compared the results with those from the full-day analysis. The results suggest that the choice of time window does not substantially affect our findings, with differences in relative effects remaining below 1% (Supplementary Fig. S14).

## Supplementary information

Supplementary Information

## Data Availability

Air quality data at London and other cities can be retrieved from the Automatic Urban and Rural Network (AURN): https://uk-air.defra.gov.uk/networks/network-info?view=aurn, and London Air Quality Network (LAQN): https://www.londonair.org.uk/london/asp/datadownload.asp. Meteorological data can be accessed at NOAA integrated surface database (ISD): https://www.ncei.noaa.gov/products/land-based-station/integrated-surface-database, and ERA5 database: https://cds.climate.copernicus.eu/datasets/reanalysis-era5-single-levels?tab=download.

## Code availability

Code for Machine learning-based Weather Normalisation and Augmented Synthetic Control Method (ASCM) are accessible at https://github.com/clnair-ascm/aqpet and https://github.com/tu-xuu/London_ULEZ.
