# Supplementary material for: Further improvement in London’s air quality demands more than the Ultra Low Emission Zone policy
Source: NPJ Clean Air. 2025 Oct 22;1(1):29. doi: 10.1038/s44407-025-00030-9 (PMC12545172; doi:10.1038/s44407-025-00030-9)
Supplement: Supplementary file 1 — Supplementary Information [file 44407_2025_30_MOESM1_ESM.pdf]

Further improvement in London's Air Quality Demands More Than the Ultra Low Emission Zone policy

Support Information

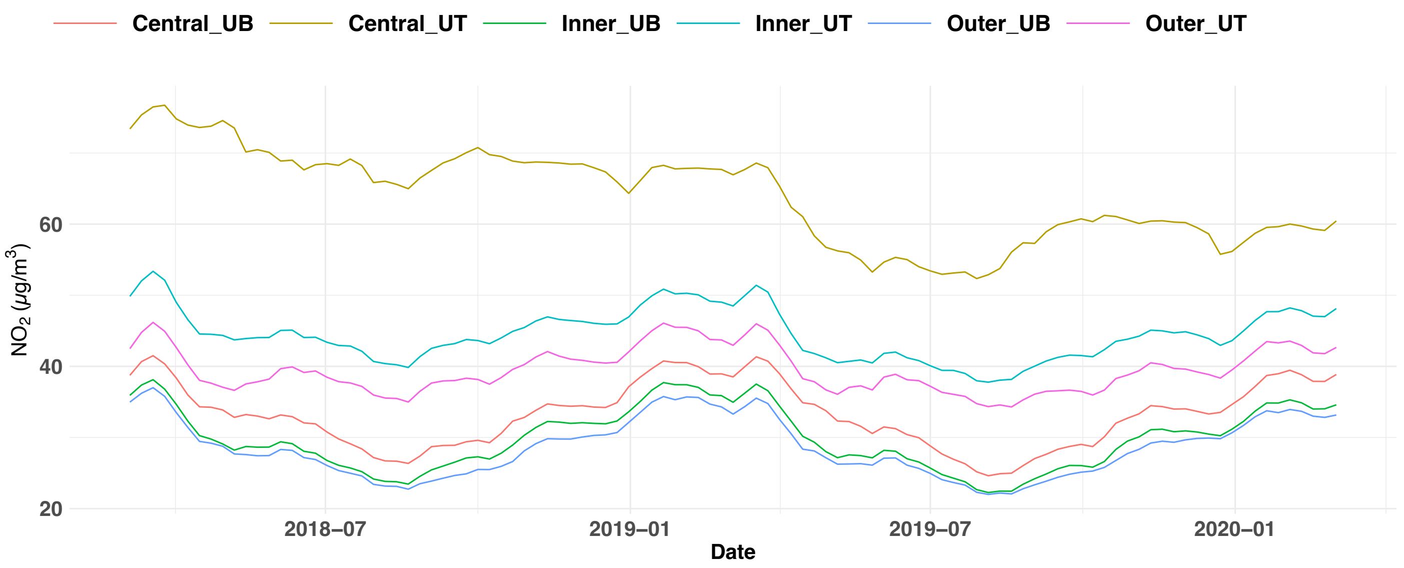

Figure S1. Weather-normalised NO_2 concentrations for urban background and urban traffic in London (2018-03-08 to 2020-03-08)
UB: Urban Background UT: Urban Traffic

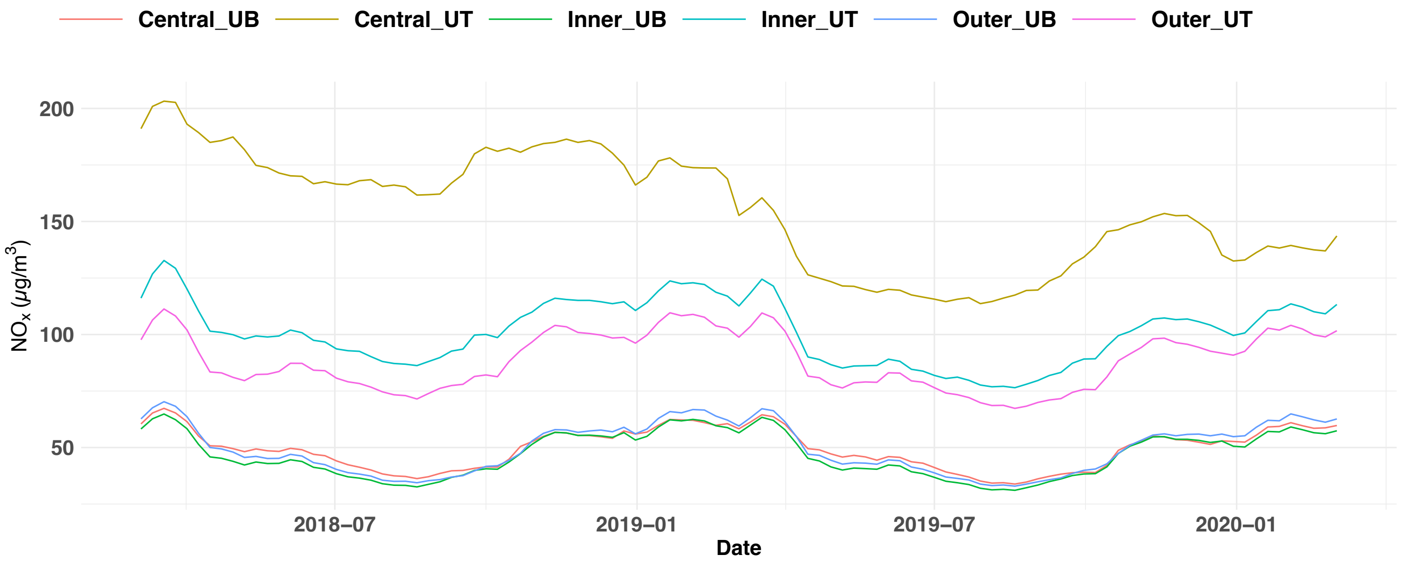

Figure S2. Weather-normalised NO_X concentrations for urban background and urban traffic in London (2018-03-08 to 2020-03-08)

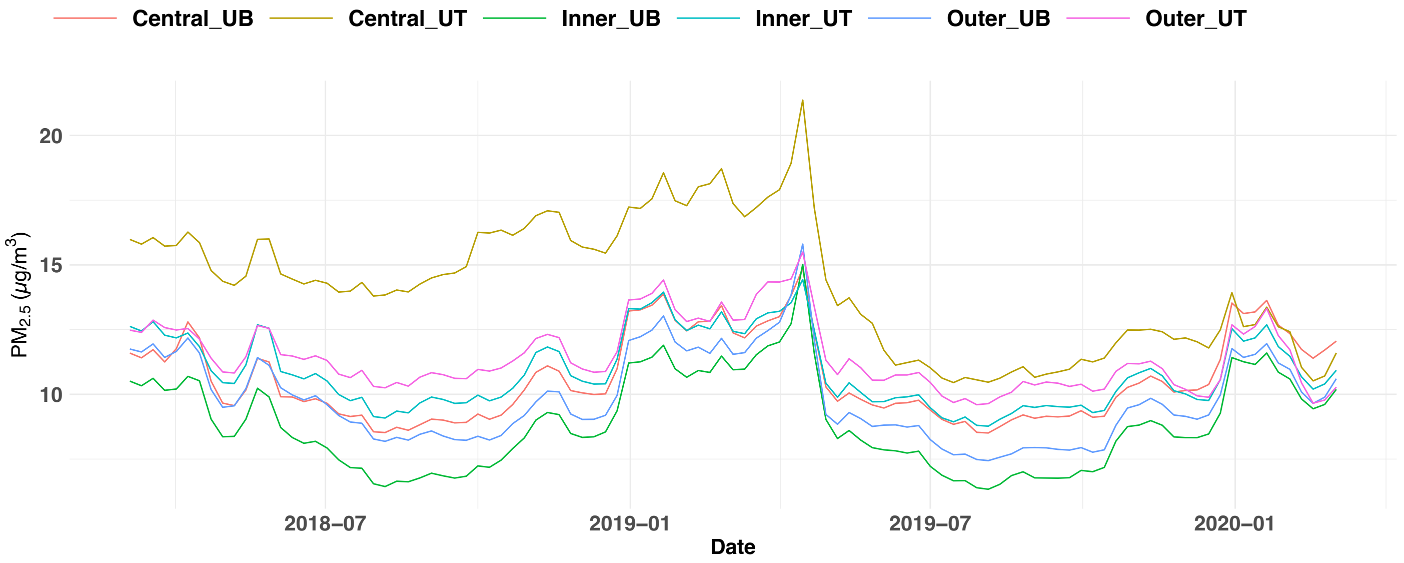

Figure S3. Weather-normalised $PM_{2.5}$ concentrations for urban background and urban traffic in London (2018-03-08 to 2020-03-08)

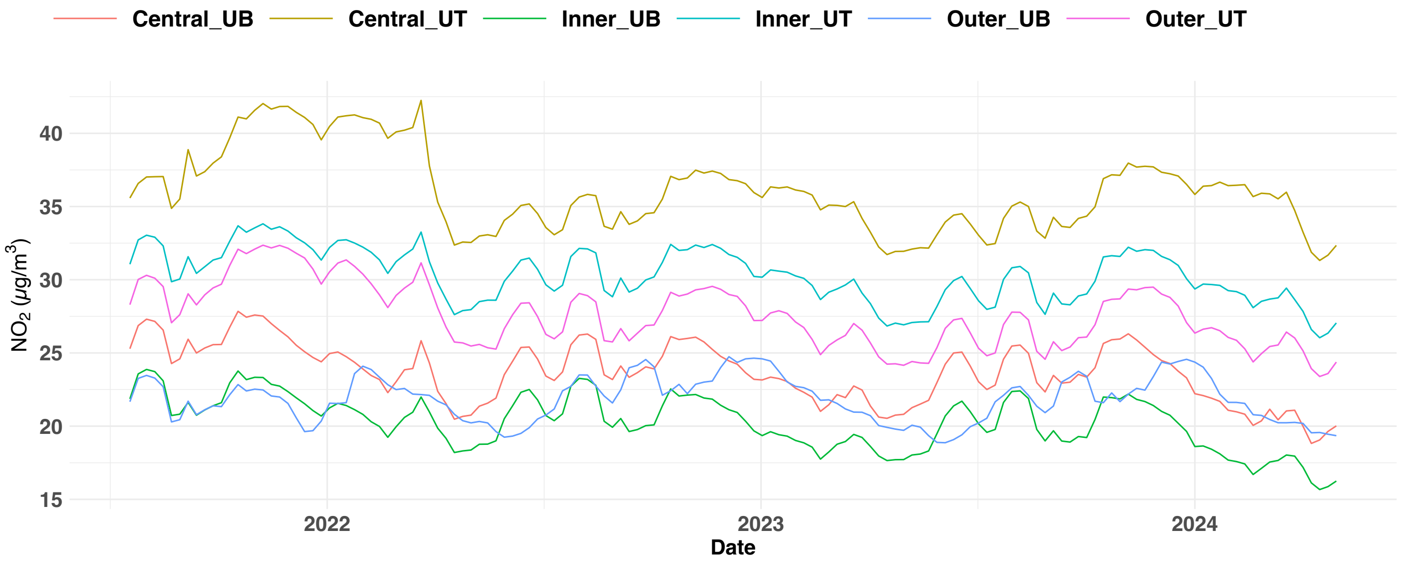

Figure S4. Weather-normalised NO_2 concentrations for urban background and urban traffic in London (2021-07-19 to 2024-04-30)

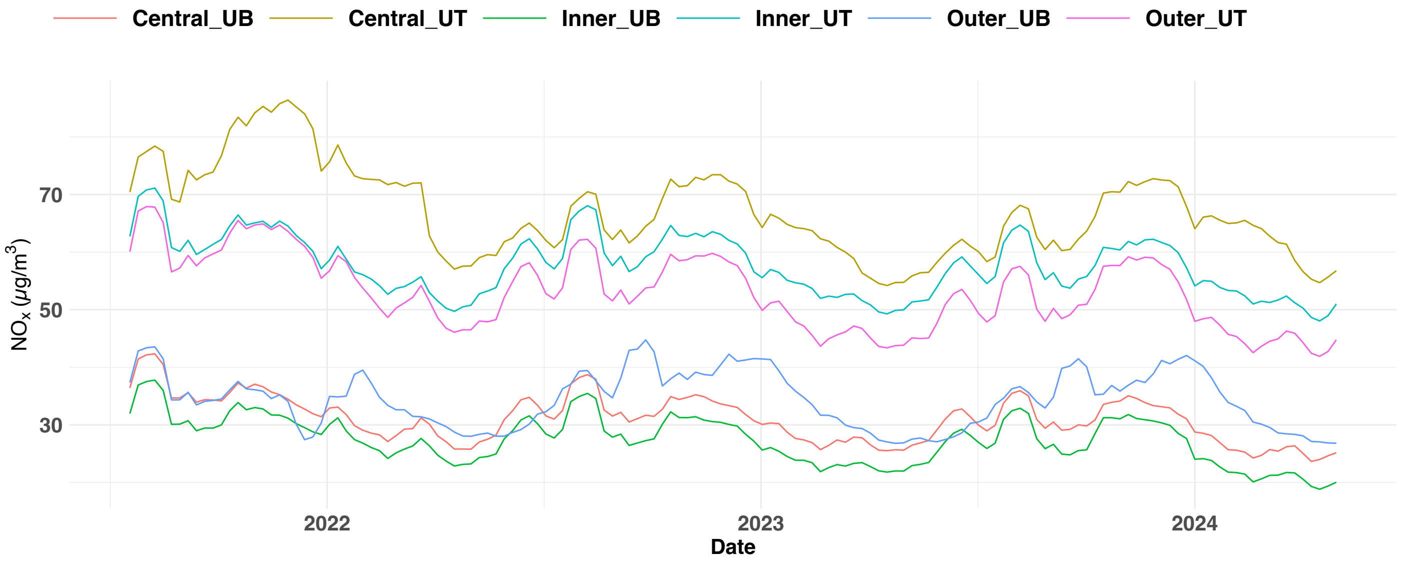

Figure S5. Weather-normalised NO_X concentrations for urban background and urban traffic in London (2021-07-19 to 2024-04-30)

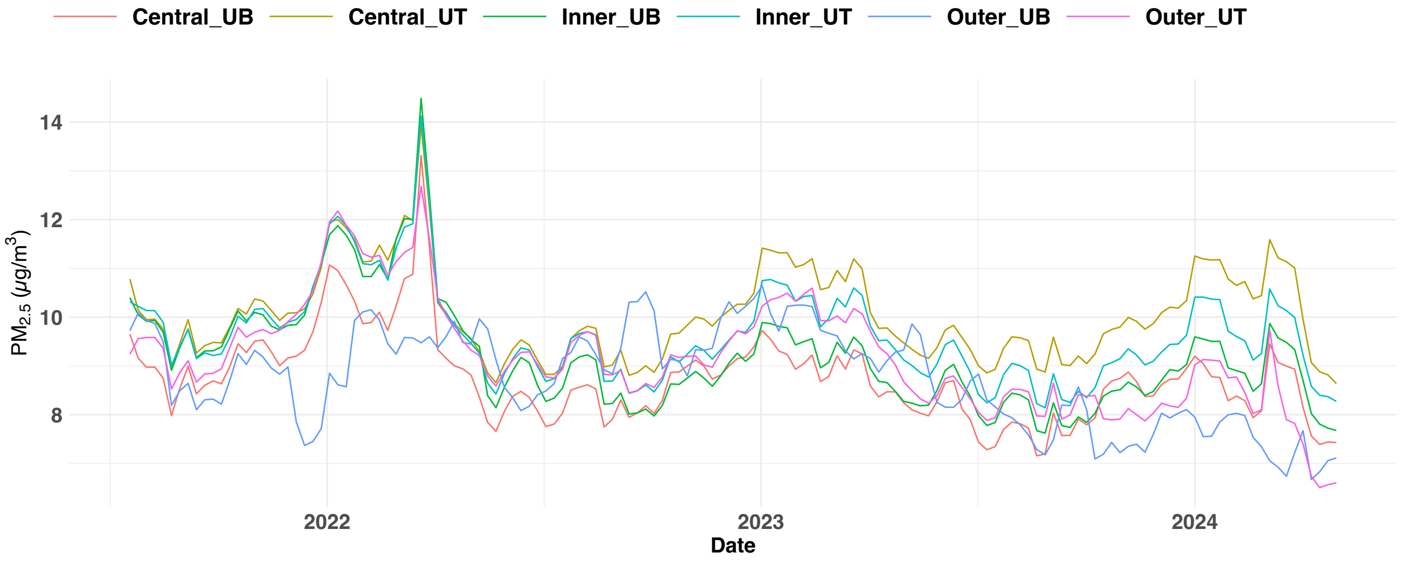

Figure S6. Weather-normalised $PM_{2.5}$ concentrations for urban background and urban traffic in London (2021-07-19 to 2024-04-30)

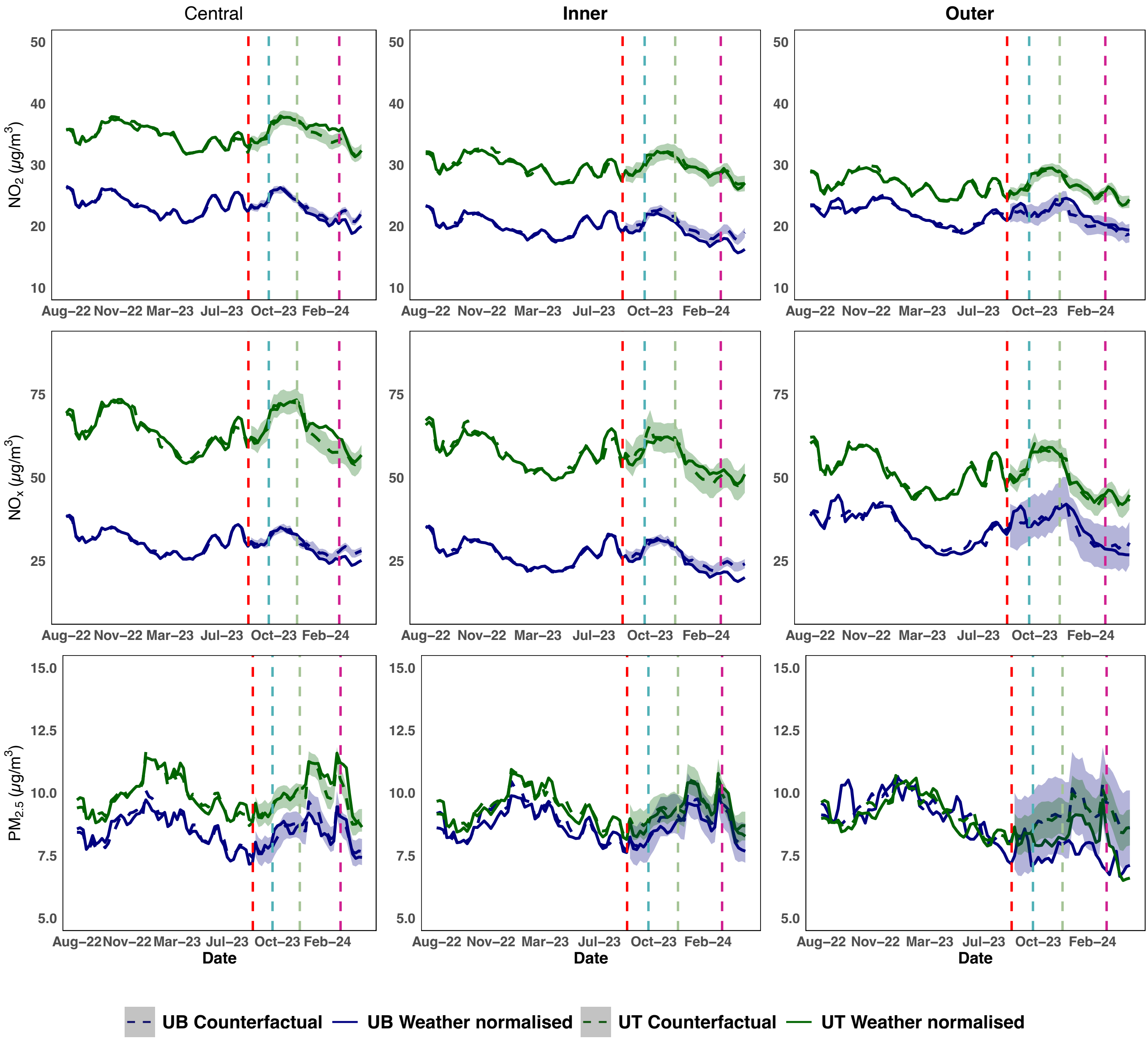

Figure S7. Weather normalised and counterfactual concentrations of NO_2, NO_X and $PM_{2.5}$ at urban background and traffic sites in central, inner and outer London (see Fig. 1 for a map), respectively, following the ULEZ3 implementation (UB: Urban Background UT: Urban Traffic)

The red dotted vertical line represents the date of ULEZ3 implementation (29th August 2023) and the rest dashed lines represent one month, three months and six months after policy implementation. The blue and green trends represent the weather normalization pollution levels and their counterfactual levels, respectively. The green shaded area represents the 95% point-wise confidence intervals.

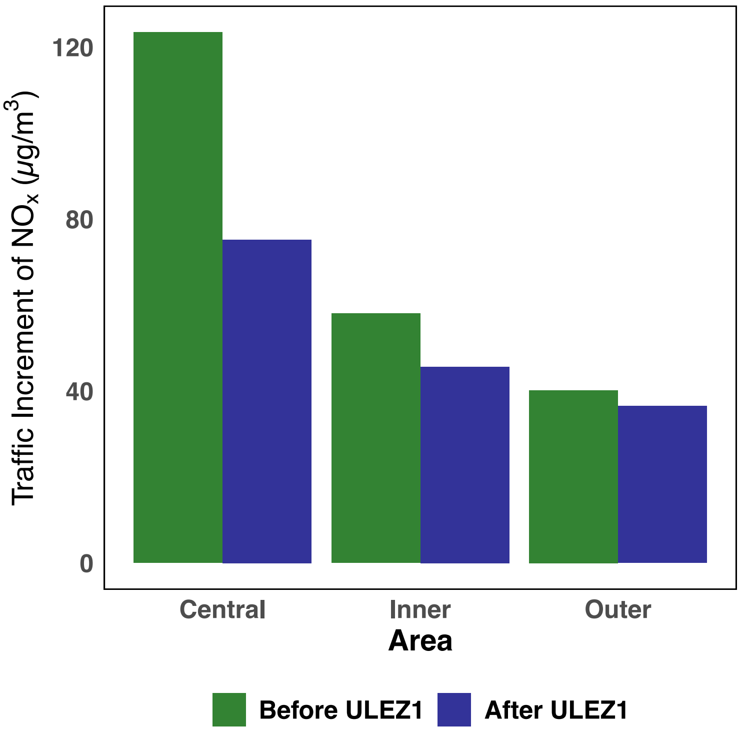

Figure S8. Traffic Increment of NO_X for central, inner, and outer London, before and after the implementation of ULEZ1

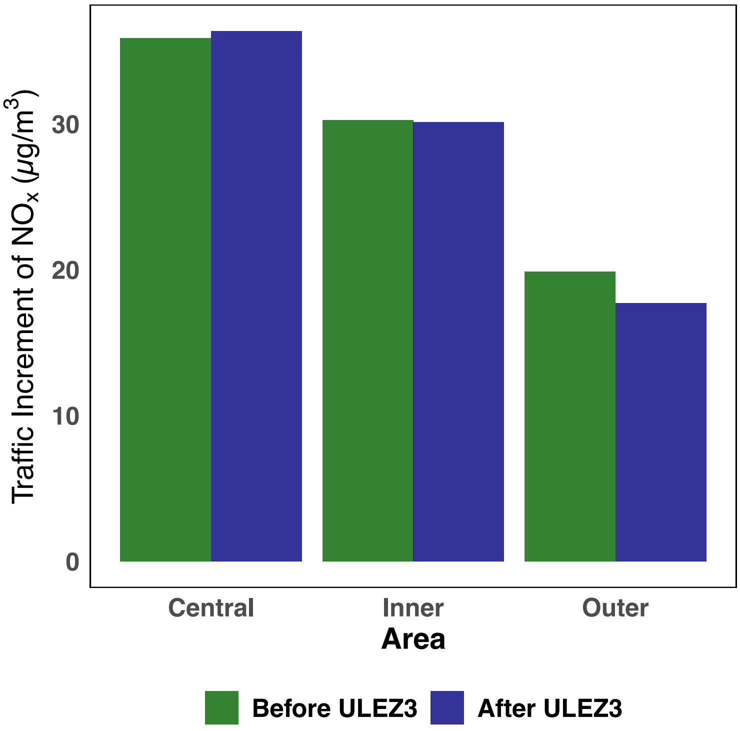

Figure S9. Traffic Increment of NO_X for central, inner, and outer London, before and after the implementation of ULEZ3

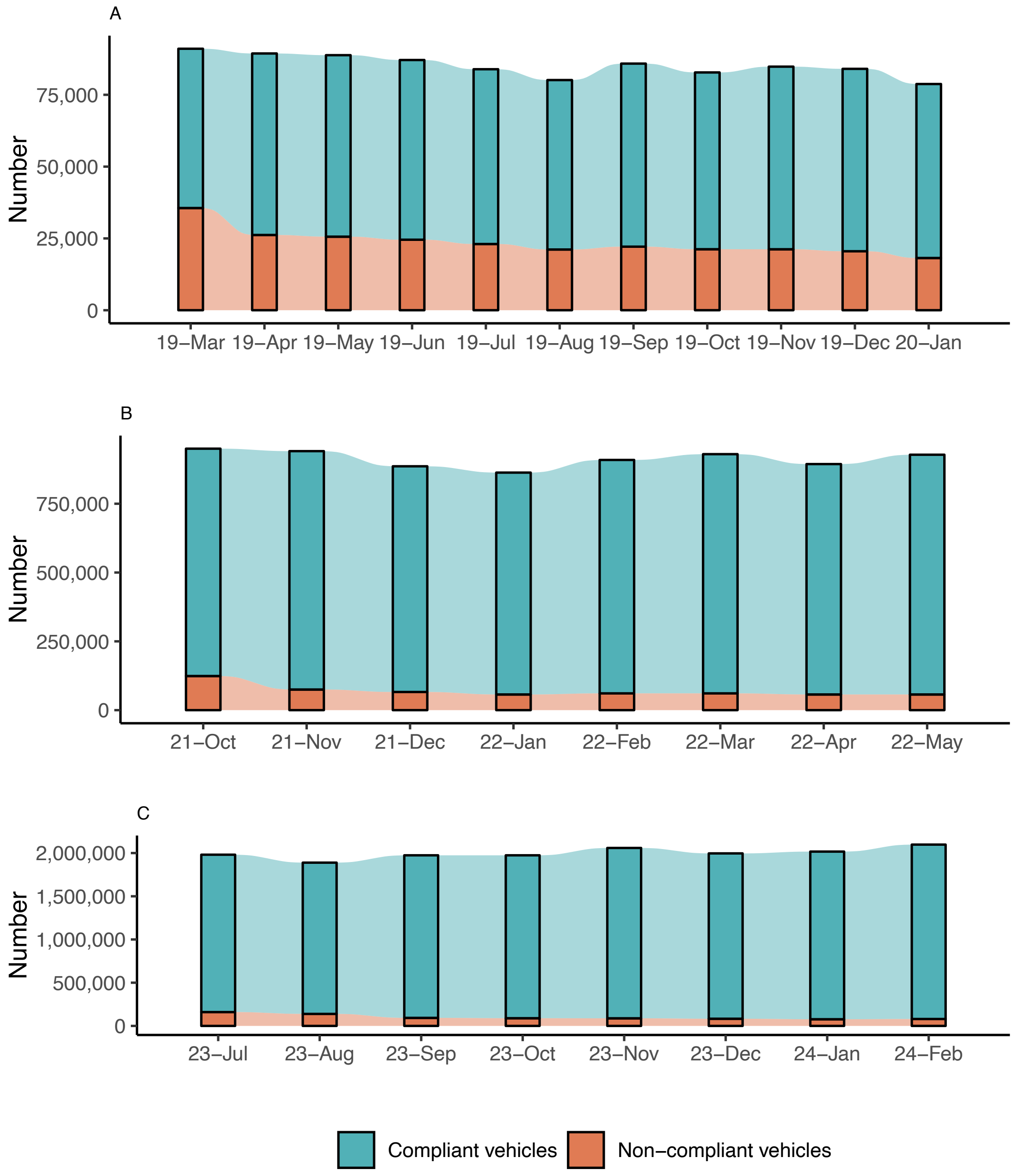

Figure S10. Numbers of Unique compliant vehicles within the ULEZ areas.

A. Congestion Charge hours (7:00-18:00, Monday to Friday) Numbers of Unique Compliant Vehicles within central London; B. 24 hours Proportion of Unique Compliant Vehicles within central and inner London; C. 24 hours Numbers of Unique Compliant Vehicles within whole London

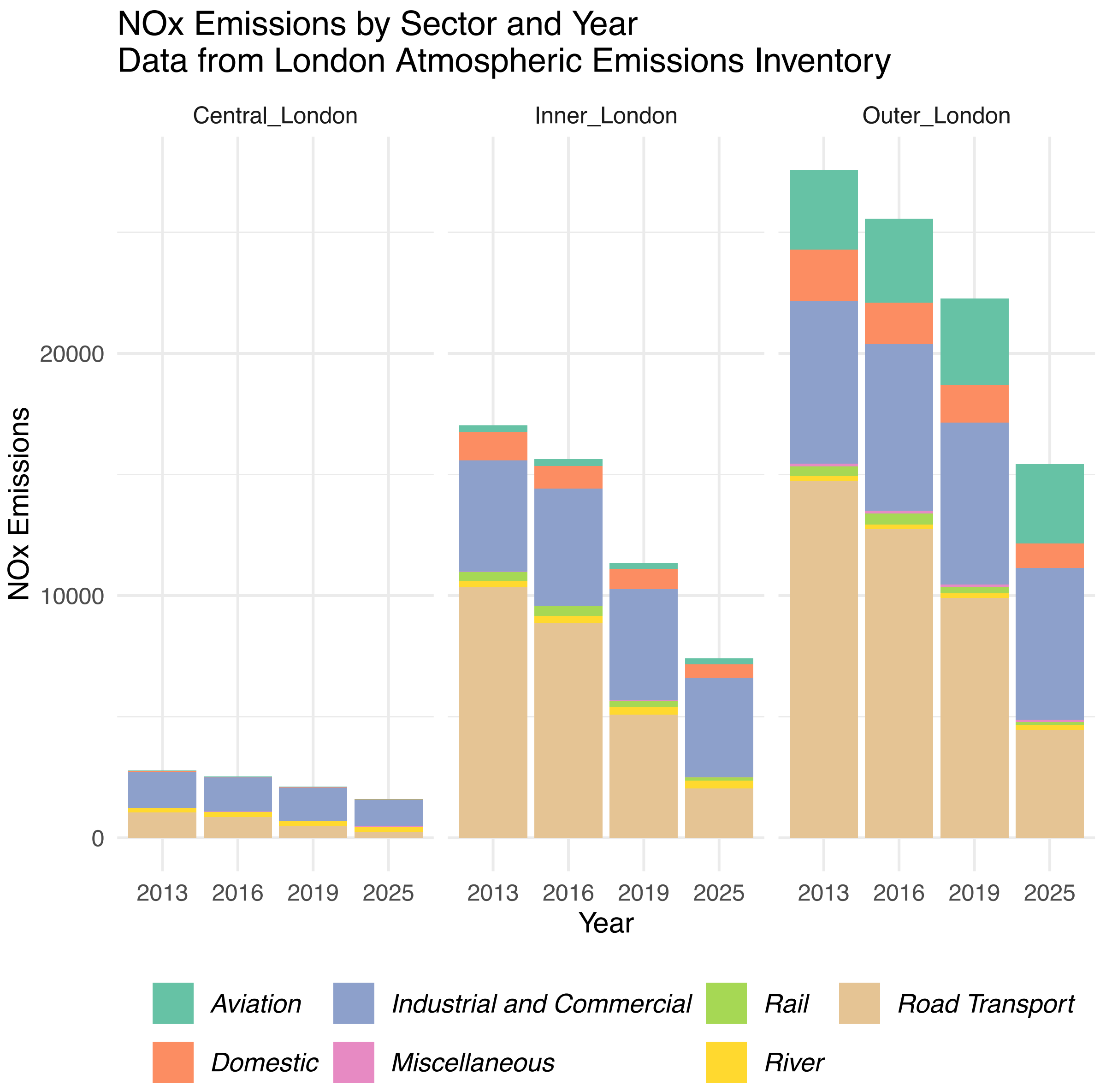

Figure S11. NO_X Emissions by years and sectors in London

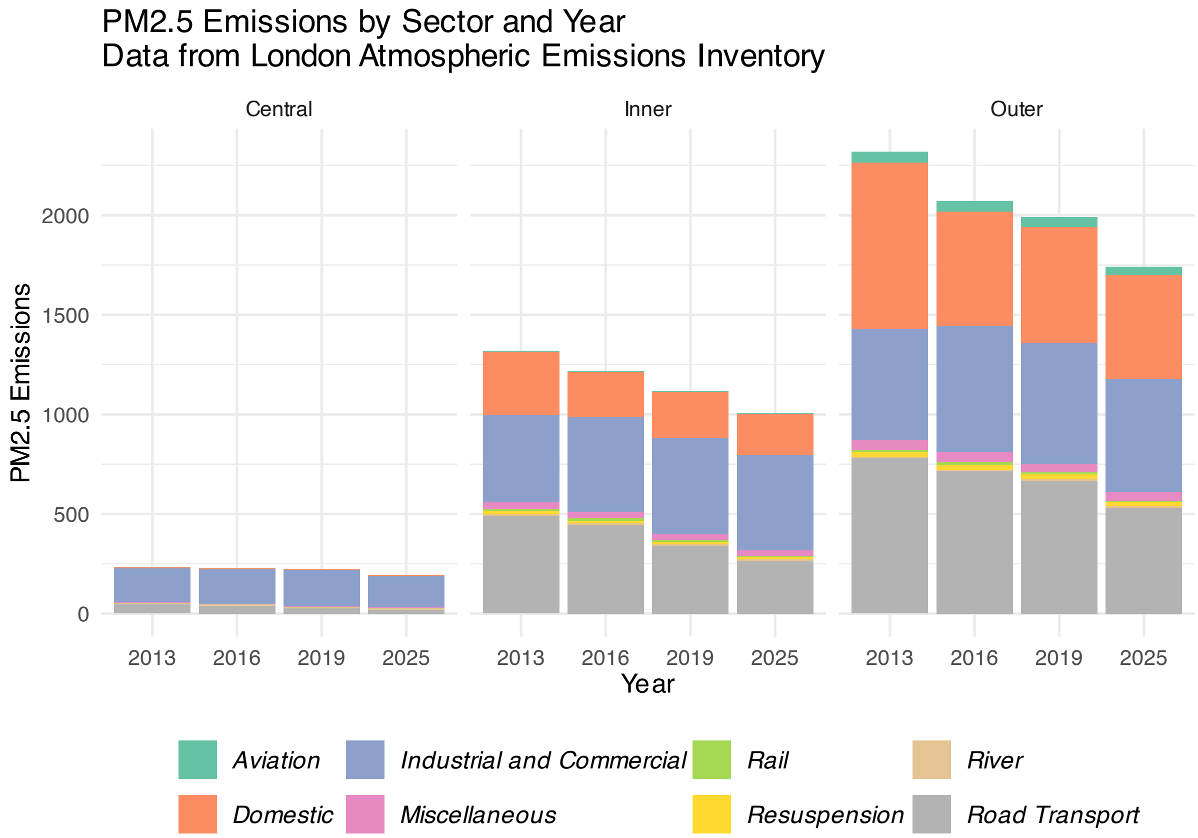

Figure S12. PM$_{2.5}$ Emissions by years and sectors in London

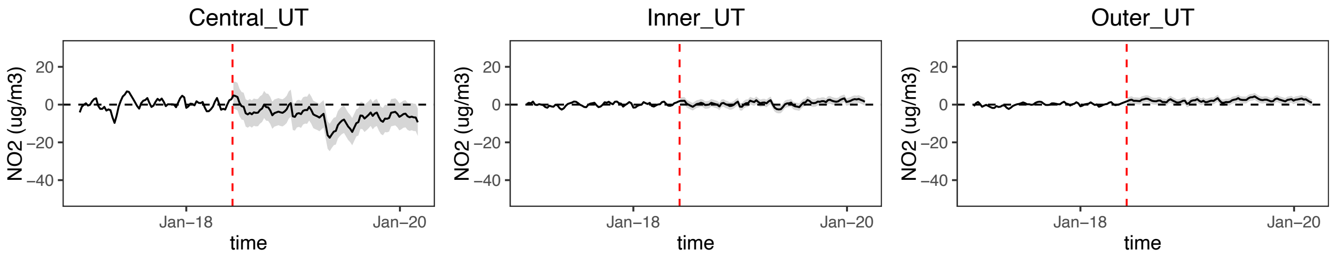

UT: Urban Traffic

Figure S13. Announcement effect results on 2018-06-08 (Time placebo test).

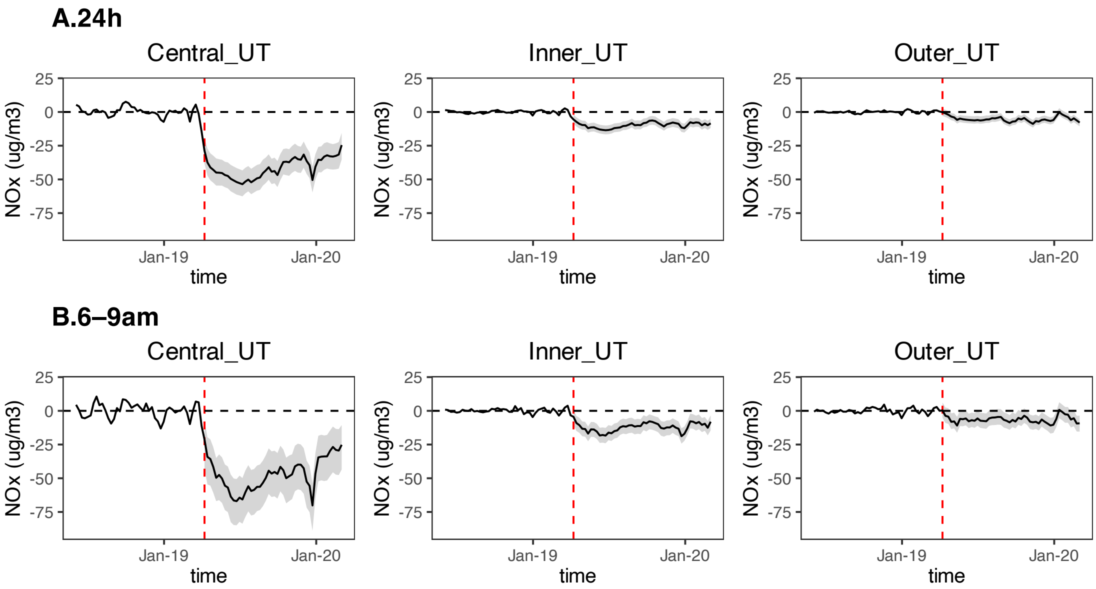

Figure S14. Policy effect on NO_X in different areas for Urban Traffic sites in different time period after ULEZ1. A. 24-hour results (main results) B. 6-9am (peak hour results).

The estimated effects were not significantly different between the two time periods (about 1% difference), the model performance and fit were generally better when using full-day data. Since the ULEZ policy operates 24 hours a day with the aim of improving air quality across all hours, using 24-hour data likely provides a more representative assessment of the policy's impact. Moreover, according to data from Transport for London (TfL), traffic volumes on major roads such as Marylebone Road [1] in central London, where pollution levels are among the highest, show relatively small differences between peak and off-peak hours. UT: Urban Traffic

Table S1. Treatment Sites Information

Code	Site	Site type	Latitude	Longitude
CA1	Camden Kerbside	UT	51.54421	-0.175269
EA8	Ealing Horn Lane	UT	51.51895	-0.265617
HG1	Haringey Roadside	UT	51.5993	-0.068218
CLL2	London Bloomsbury	UB	51.52229	-0.125889
HG4	London Haringey Priory Park South	UB	51.584128	-0.125254
HR3	London Harrow Stanmore	UB	51.617333	-0.298777
HIL	London Hillingdon	UB	51.49633	-0.460861
HP1	London Honor Oak Park	UB	51.449674	-0.037418
MY1	London Marylebone Road	UT	51.52253	-0.154611
KC1	London N. Kensington	UB	51.52105	-0.213492
TED2	London Teddington Bushy Park	UB	51.425286	-0.345606
HORS	London Westminster	UB	51.49467	-0.131931
SK5	Southwark A2 Old Kent Road	UT	51.480499	-0.05955
TH2	Tower Hamlets Roadside	UT	51.52253	-0.042155
CTA	City of London - Bell Wharf Lane	UT	51.5105969	-0.0921029
SKC	Southwark - South Circular Road	UT	51.442633	-0.077654
SK8	Southwark - Tower Bridge Road	UT	51.5013907	-0.0782034
SK9	Southwark - A2 Old Kent Road FIDAS	UT	51.4804995	-0.0595529
SKA	Southwark - Lower Road	UT	51.4965604	-0.0530504

SKB	Southwark - Vicarage Grove	UT	51.4735803	-0.0877819
CE2	Waterloo Place (The Crown Estate)	UT	51.5075819	-0.1330684
TL4	Greenwich - Tunnel Avenue	UT	51.495057	0.00410747
TL5	Newham - Hoola Tower	UT	51.508198	0.01500215
TL6	Newham - Britannia Gate	UT	51.503858	0.02057442
NM4	Newham - East Ham Town Hall	UT	51.5330166	0.05500021
ST9	Sutton - Beddington Village	UT	51.3712011	-0.1319488
GV2	Westminster - Duke Street (Grosvenor)	UT	51.5129999	-0.1509135
CW3	Tower Hamlets - Jubilee Park	UB	51.5029879	-0.018022
HK9	Hackney - Queensbridge Road	UT	51.539324	-0.070079
HN1	Hackney - Homerton Library	UT	51.5487472	-0.0418807
HN0	Hackney - Amhurst Road	UT	51.550056	-0.0633016
LW6	Lewisham - Laurence House Catford	UT	51.4447808	-0.0221832
CT9	City of London - Guildhall	UB	51.515907	-0.092024
LW5	Lewisham - Deptford	UB	51.4795313	-0.0252606
CE3	Regent Street (The Crown Estate)	UT	51.5113771	-0.1392494
WMD	Westminster - Elizabeth Bridge	UT	51.4922482	-0.1471148
CT6	City of London - Walbrook Wharf	UT	51.510499	-0.091634
ST4	Sutton - Wallington	UT	51.3586596	-0.1497239
CT8	City of London - Upper Thames Street	UT	51.5095437	-0.0873572
WA2	Wandsworth - Wandsworth Town Hall	UB	51.456962	-0.191074
HV1	Havering - Rainham	UT	51.5207875	0.20546071
HV3	Havering - Romford	UT	51.572976	0.179079
BT4	Brent - Ikea	UT	51.552476	-0.258089
WM5	Westminster - Covent Garden	UB	51.511977	-0.1216272
LB4	Lambeth - Brixton Road	UT	51.4641135	-0.114581
MY7	Westminster - Marylebone Road FDMS	UT	51.52254	-0.15459
EN5	Enfield - Bowes Primary School	UT	51.613865	-0.125338
BL0	Camden - Bloomsbury	UB	51.522287	-0.125848
RB4	Redbridge - Gardner Close	UT	51.57661	0.030858
RI1	Richmond Upon Thames - Castelnau	UT	51.480189	-0.237335
CR5	Croydon - Norbury	UT	51.411349	-0.12311
EN4	Enfield - Derby Road	UT	51.614864	-0.0507658
GB6	Greenwich - Falconwood	UT	51.4563	0.085606
RHG	Richmond Upon Thames - Chertsey Road	UT	51.4531423	-0.3412181
GR7	Greenwich - Blackheath	UT	51.472504	-0.012381
HK6	Hackney - Old Street	UT	51.526454	-0.08491
HR1	Harrow - Stanmore	UB	51.617327	-0.298775
HR2	Harrow - Pinner Road	UT	51.588417	-0.362989
EA6	Ealing - Hanger Lane Gyratory	UT	51.53085	-0.292488
GR8	Greenwich - Woolwich Flyover	UT	51.486884	0.017901
GV1	Westminster - Ebury Street (Grosvenor)	UT	51.493492	-0.149906
BQ8	Bexley - Belvedere West FDMS	UB	51.4946487	0.13727911

CR7	Croydon - Purley Way A23	UT	51.3622306	-0.1176045
CT2	City of London - Farringdon Street	UT	51.5145253	-0.1045156
CT4	City of London - Beech Street	UT	51.5202253	-0.096106
EI1	Ealing - Western Avenue	UT	51.5236078	-0.2655026
GB0	Greenwich - Falconwood FDMS	UT	51.4563	0.085606
WA7	Wandsworth - Putney High Street	UT	51.463429	-0.215871
NM2	Newham - Cam Road	UT	51.537598	-0.002138
LW4	Lewisham - Loampit Vale	UT	51.4646911	-0.0160682
MR8	Marylebone Road - BAM	UT	51.5225094	-0.1546222
KT5	Kingston Upon Thames - Cromwell Road	UT	51.412308	-0.2965848
KT6	Kingston Upon Thames - Kingston Vale	UT	51.4355007	-0.2570299
WMC	Westminster - Cavendish Square	UT	51.5168016	-0.1456573
GN6	Greenwich - John Harrison Way	UT	51.4937747	0.01077962
IM1	Camden - Holborn (Bee Midtown)	UT	51.5173675	-0.1201947
SK6	Southwark - Elephant and Castle	UB	51.4931557	-0.101527
WM9	Westminster - Victoria (Victoria BID)	UT	51.4977332	-0.1442414
LH0	Hillingdon - Harlington	UB	51.48878	-0.441627
WM0	Westminster - Horseferry Road	UB	51.494681	-0.131938
GR9	Greenwich - Westhorne Avenue	UT	51.456357	0.040725
KC7	Kensington and Chelsea - North Ken FDMS	UB	51.5210467	-0.2134921
BQ7	Bexley - Belvedere West	UB	51.4946487	0.13727911
BY7	Bromley - Harwood Avenue	UT	51.405546	0.018882
CD1	Camden - Swiss Cottage	UT	51.544219	-0.175284
IS2	Islington - Holloway Road	UT	51.555378	-0.116146
KC2	Kensington and Chelsea - Cromwell Road	UT	51.4955039	-0.1788095
KC3	Kensington and Chelsea - Knightsbridge	UT	51.4991395	-0.1643376
KC4	Kensington and Chelsea - Kings Road	UT	51.4874369	-0.1683971
KC5	Kensington and Chelsea - Earls Court Rd	UT	51.4901976	-0.1908633
LW1	Lewisham - Catford	UB	51.445468	-0.020266
LW2	Lewisham - New Cross	UT	51.474954	-0.039641
BT6	Brent - John Keble Primary School	UT	51.537799	-0.247793
GN3	Greenwich - Plumstead High Street	UT	51.486957	0.095111
GN4	Greenwich - Fiveways Sidcup Rd A20	UT	51.4346627	0.06422247
GN0	Greenwich - A206 Burrage Grove	UT	51.490532	0.074003
TH4	Tower Hamlets - Blackwall	UT	51.5150462	-0.0084185
CT3	City of London - The Aldgate School	UB	51.5138472	-0.0777657
IS6	Islington - Arsenal	UB	51.557895	-0.106989
WAA	Wandsworth - Battersea	UT	51.4794395	-0.141787
HF4	Hammersmith and Fulham - Shepherds Bush	UT	51.5045626	-0.22467
ME2	Merton - Merton Road	UT	51.4161385	-0.1922308
CD9	Camden - Euston Road	UT	51.527975	-0.1287742
EN7	Enfield - Prince of Wales School	UB	51.6686433	-0.0220072
WM6	Westminster - Oxford Street	UT	51.5139287	-0.1527927

WA9	Wandsworth - Putney	UB	51.4650325	-0.2158246
WA8	Wandsworth - Putney High Street Facade	UT	51.4637206	-0.2158901
WAB	Wandsworth - Tooting High Street	UT	51.429331	-0.166524
NM3	Newham - Wren Close	UB	51.514727	0.014554
LB6	Lambeth - Streatham Green	UB	51.4282131	-0.1318686
RB7	Redbridge - Ley Street	UB	51.5694843	0.08290747
CR8	Croydon - Norbury Manor	UB	51.410039	-0.127523
KT4	Kingston Upon Thames - Tolworth Broadway	UT	51.379312	-0.281259
BT8	Brent - ARK Franklin Primary Academy	UT	51.5324055	-0.2177189
KF1	Kensington and Chelsea - North Ken FIDAS	UB	51.5210467	-0.2134921
WMB	Westminster - Oxford Street East	UT	51.516066	-0.1351639
NB1	Westminster - Strand (Northbank BID)	UT	51.5119701	-0.1167131
WAC	Wandsworth - Lavender Hill (Clapham Jct)	UT	51.4636904	-0.1667135
CR9	Croydon - Park Lane	UT	51.3739531	-0.0967638
ME9	Merton - Morden Civic Centre 2	UT	51.40162	-0.1958921
GN5	Greenwich - Trafalgar Road (Hoskins St)	UT	51.4839073	0.0004074
CE1	Regent Street facade (The Crown Estate)	UT	51.5111825	-0.1391141
EI3	Ealing - Acton Vale	UB	51.5038528	-0.2546681
WMA	Westminster - Buckingham Palace Road	UT	51.4932328	-0.1473918

UB: Urban Background UT: Urban Traffic

Table S2. Control Sites Information

Code	Site	Site type	Latitude	Longitude
ABD	Aberdeen	UB	57.14	-2.11
ABD0	Aberdeen Market Street 2	UT	57.14	-2.09
ABD7	Aberdeen Union Street Roadside	UT	57.14	-2.11
ARM6	Armagh Roadside	UT	57.35	-6.65
BAR3	Barnsley Gawber	UB	53.56	-1.51
BEL2	Belfast Centre	UB	54.6	-5.93
BIRR	Birmingham A4540 Roadside	UT	52.48	-1.88
AGRN	Birmingham Acocks Green	UB	52.44	-1.83
BIR1	Birmingham Tyburn	UB	52.51	-1.83
BLAR	Blackburn Accrington Road	UB	53.75	-2.45
BLC2	Blackpool Marton	UB	53.8	-3.01
BORN	Bournemouth	UB	50.74	-1.83
BDMA	Bradford Mayo Avenue	UB	53.77	-1.76
BRT3	Brighton Preston Park	UB	50.84	-0.15
BRS8	Bristol St Paul	UB	51.46	-2.58
BR11	Bristol Temple Way	UT	51.46	-2.58
BURW	Bury Whitefield Roadside	UT	53.56	-2.29
CAM3	Cambridge Gonville Place	UT	52.20	0.13
CAM	Cambridge Roadside	UT	52.2	0.12
CANT	Canterbury	UB	51.27	1.1
CARD	Cardiff Centre	UB	51.48	-3.18
CHP	Chepstow A48	UT	51.64	-2.68
DCST	Doncaster A630 Cleveland Street	UT	53.52	-1.14
DUMF	Dumfries	UT	55.07	-3.61
ED1	Edinburgh St John's Road	UT	55.94	-3.28
ED3	Edinburgh St Leonards	UB	55.95	-3.18
EX	Exeter Roadside	UT	50.73	-3.53
GA3	Gateshead Lychgate Court	UT	54.96	-1.60
GGWR	Glasgow Great Western Road	UT	55.87	-4.27
GHSR	Glasgow High Street	UT	55.86	-4.24
GLA4	Glasgow Kerbside	UT	55.86	-4.26
GLKP	Glasgow Townhead	UB	55.86	-4.24
HG1	Haringey Roadside	UT	51.6	-0.07
HUL2	Hull Freetown	UB	53.75	-0.34
LEAM	Leamington Spa	UB	52.29	-1.53
LEAR	Leamington Spa Rugby Road	UT	52.29	-1.54
LED6	Leeds Headingley Kerbside	UT	53.82	-1.58
LEIR	Leicester_A594_Roadside	UT	52.64	-1.12
LEIC	Leicester Centre	UB	52.63	-1.13
LECU	Leicester University	UB	52.62	-1.13
LUTR	Luton A505 Roadside	UT	51.89	-0.46

HB007	Luton Dunstable Road East	UT	51.88	-0.42
MAN3	Manchester Piccadilly	UB	53.48	-2.24
NEWC	Newcastle Centre	UB	54.98	-1.61
NCA3	Newcastle Cradlewell Roadside	UT	54.99	-1.6
NOTT	Nottingham Centre	UB	52.95	-1.15
OX8	Oxford St Ebbes	UB	51.74	-1.26
PLYM	Plymouth Centre	UB	50.37	-4.14
PRES	Preston	UB	53.77	-2.68
REA1	Reading New Town	UB	51.45	-0.94
SHBR	Sheffield Barnsley Road	UT	53.40	-1.46
SHE	Sheffield Tinsley	UB	53.41	-1.4
SOUT	Southampton Centre	UB	50.91	-1.4
STOK	Stoke on Trent Centre	UB	53.03	-2.18
SUN2	Sunderland Silksworth	UB	54.88	-1.41
SWA5	Swansea Morriston Roadside	UT	51.66	-3.92
SWA1	Swansea Roadside	UT	51.53	-3.95
WIG5	Wigan Centre	UB	53.55	-2.64
TRAN	Wirral Tranmere	UB	53.37	-3.02
YK11	York Fishergate	UT	53.95	-1.08

Table S3. NO_2, NO_X, $PM_{2.5}$ Average absolute causal effect at urban background and traffic site for ULEZ1 post one, three, six, nine months and eleven months for ULEZ1

Pollutants	Urban Background						Urban Traffic					
μg m^{-3}	Central		Inner		Outer		Central		Inner		Outer	
NO_2	1 month	-1.16 (-1.87, -0.43)	1 month	-1.64 (-2.44, -0.82)	1 month	-1.27 (-2.18, -0.42)	1 month	-13.24 (-17.39, -8.7)	1 month	-2.36 (-3.17, -1.58)	1 month	-0.16 (-0.94, 0.63)
	3 months	-2.74 (-3.51, -1.92)	3 months	-2.52 (-3.26, -1.7)	3 months	-1.25 (-2.14, -0.4)	3 months	-13.33 (-17.53, -8.89)	3 months	-2.34 (-3.14, -1.56)	3 months	-0.56 (-1.32, 0.26)
	6 months	-1.98 (-2.79, -1.2)	6 months	-1.78 (-2.58, -0.96)	6 months	-0.6 (-1.49, 0.22)	6 months	-11.61 (-15.88, -7.19)	6 months	-1.28 (-2.13, -0.53)	6 months	0.09 (-0.69, 0.87)
	9 months	-1.2 (-2.02, -0.42)	9 months	-1.25 (-2.07, -0.43)	9 months	-0.21 (-1.11, 0.6)	9 months	-12.34 (-16.68, -7.88)	9 months	-1.34 (-2.21, -0.61)	9 months	0.06 (-0.77, 0.81)
	11 months	-0.99 (-1.81, -0.2)	11 months	-1.17 (-2, -0.35)	11 months	-0.2 (-1.1, 0.61)	11 months	-12.41 (-16.77, -7.94)	11 months	-1.43 (-2.31, -0.7)	11 months	-0.05 (-0.89, 0.69)
NO_X	1 month	-1.26 (-2.85, 0.46)	1 month	-1.88 (-3.9, 0.08)	1 month	-1.46 (-4.24, 1.4)	1 month	-43.86 (-53.4, -33.59)	1 month	-10.34 (-13.67, -7.05)	1 month	-4.05 (-6.73, -1.38)
	3 months	-3.08 (-4.66, -1.36)	3 months	-2.72 (-4.77, -0.79)	3 months	-1.87 (-4.5, 0.97)	3 months	-48.14 (-57.38, -37.91)	3 months	-11.79 (-14.94, -8.36)	3 months	-5.24 (-7.79, -2.37)
	6 months	-2.63 (-4.23, -0.95)	6 months	-2.15 (-4.11, -0.15)	6 months	-1.21 (-3.89, 1.51)	6 months	-46.47 (-55.69, -36.39)	6 months	-10.37 (-13.51, -6.95)	6 months	-5.44 (-8.03, -2.59)
	9 months	-2.01 (-3.64, -0.34)	9 months	-2.2 (-4.13, -0.13)	9 months	-1.24 (-3.96, 1.44)	9 months	-43.33 (-52.71, -33.35)	9 months	-10 (-13.17, -6.59)	9 months	-5.47 (-8.06, -2.61)
	11 months	-1.89 (-3.54, -0.23)	11 months	-2.5 (-4.45, -0.43)	11 months	-1.35 (-4.08, 1.35)	11 months	-41.74 (-51.2, -31.84)	11 months	-9.84 (-13.02, -6.43)	11 months	-5.45 (-8.05, -2.61)
$PM_{2.5}$	1 month	-0.03 (-1, 0.97)	1 month	-0.09 (-1.08, 0.89)	1 month	-0.28 (-1.23, 0.61)	1 month	-0.9 (-1.73, -0.02)	1 month	-0.28 (-1.24, 0.64)	1 month	-0.14 (-1.18, 1)
	3 months	-0.04 (-1.02, 0.95)	3 months	0.03 (-0.95, 1.03)	3 months	-0.26 (-1.22, 0.67)	3 months	-1.79 (-2.6, -0.89)	3 months	-0.15 (-1.1, 0.78)	3 months	-0.1 (-1.13, 1.06)
	6 months	-0.07 (-1.1, 0.92)	6 months	0.08 (-0.87, 1.11)	6 months	-0.21 (-1.17, 0.82)	6 months	-2.21 (-3.04, -1.34)	6 months	0.02 (-0.94, 0.94)	6 months	0.04 (-1, 1.19)
	9 months	-0.07 (-1.1, 0.94)	9 months	0.23 (-0.74, 1.24)	9 months	-0.13 (-1.1, 0.88)	9 months	-2.69 (-3.5, -1.81)	9 months	-0.02 (-0.97, 0.91)	9 months	-0.17 (-1.2, 0.99)
	11 months	-0.04 (-1.08, 0.97)	11 months	0.29 (-0.69, 1.27)	11 months	-0.12 (-1.09, 0.88)	11 months	-3.1 (-3.91, -2.21)	11 months	-0.14 (-1.1, 0.78)	11 months	-0.42 (-1.44, 0.74)

Note: Post 11 months refers to pre-covid lockdown

Table S4. NO_2, NO_X, $PM_{2.5}$ Average relative causal effect urban background and traffic for ULEZ1 in post one, three, six, nine months and 11 months for ULEZ1

Pollutants	Urban Background						Urban Traffic					
µg m^{-3}	Central		Inner		Outer		Central		Inner		Outer	
	1 month	-3.41% (-5.49%, -1.25%)	1 month	-5.55% (-8.26%, -2.78%)	1 month	-4.53% (-7.74%, -1.48%)	1 month	-19.00% (-24.95%, -12.49%)	1 month	-5.44% (-7.31%, -3.64%)	1 month	-0.44% (-2.53%, 1.69%)
	3 months	-8.22% (-10.55%, -5.78%)	3 months	-8.61% (-11.13%, -5.79%)	3 months	-4.59% (-7.89%, -1.47%)	3 months	-19.57% (-25.73%, -13.05%)	3 months	-5.44% (-7.30%, -3.62%)	3 months	-1.50% (-3.50%, 0.68%)
NO_2	6 months	-6.38% (-8.98%, -3.87%)	6 months	-6.47% (-9.35%, -3.48%)	6 months	-2.34% (-5.83%, 0.87%)	6 months	-17.11% (-23.39%, -10.59%)	6 months	-3.07% (-5.09%, -1.26%)	6 months	0.24% (-1.90%, 2.37%)
	9 months	-3.74% (-6.30%, -1.30%)	9 months	-4.33% (-7.19%, -1.49%)	9 months	-0.77% (-4.13%, 2.25%)	9 months	-17.75% (-23.99%, -11.34%)	9 months	-3.10% (-5.12%, -1.40%)	9 months	0.16% (-2.04%, 2.15%)
	11 months	-3.00% (-5.50%, -0.61%)	11 months	-3.94% (-6.73%, -1.18%)	11 months	-0.72% (-3.98%, 2.20%)	11 months	-17.75% (-23.99%, -11.36%)	11 months	-3.25% (-5.24%, -1.59%)	11 months	-0.13% (-2.32%, 1.80%)
	1 month	-2.62% (-5.93%, 0.95%)	1 month	-4.35% (-9.01%, 0.18%)	1 month	-3.21% (-9.33%, 3.07%)	1 month	-26.41% (-32.16%, -20.23%)	1 month	-10.66% (-14.10%, -7.27%)	1 month	-4.90% (-8.15%, -1.67%)
	3 months	-6.58% (-9.94%, -2.90%)	3 months	-6.48% (-11.36%, -1.89%)	3 months	-4.32% (-10.38%, 2.24%)	3 months	-28.81% (-34.34%, -22.69%)	3 months	-12.20% (-15.46%, -8.65%)	3 months	-6.30% (-9.36%, -2.84%)
NO_X	6 months	-6.05% (-9.72%, -2.19%)	6 months	-5.40% (-10.34%, -0.38%)	6 months	-2.99% (-9.58%, 3.71%)	6 months	-27.47% (-32.92%, -21.51%)	6 months	-10.94% (-14.25%, -7.33%)	6 months	-6.71% (-9.90%, -3.20%)
	9 months	-4.26% (-7.74%, -0.73%)	9 months	-4.91% (-9.21%, -0.29%)	9 months	-2.71% (-8.60%, 3.14%)	9 months	-25.03% (-30.44%, -19.26%)	9 months	-9.90% (-13.04%, -6.53%)	9 months	-6.26% (-9.21%, -2.98%)
	11 months	-3.87% (-7.25%, -0.47%)	11 months	-5.32% (-9.46%, -0.92%)	11 months	-2.79% (-8.42%, 2.79%)	11 months	-24.16% (-29.64%, -18.43%)	11 months	-9.50% (-12.58%, -6.21%)	11 months	-6.05% (-8.94%, -2.90%)
	1 month	-0.33% (-9.52%, 9.23%)	1 month	-0.95% (-11.65%, 9.61%)	1 month	-2.83% (-12.36%, 6.13%)	1 month	-5.87% (-11.31%, -0.13%)	1 month	-2.57% (-11.31%, 5.80%)	1 month	-1.20% (-10.10%, 8.53%)
	3 months	-0.37% (-10.44%, 9.66%)	3 months	0.36% (-11.94%, 12.83%)	3 months	-2.83% (-13.35%, 7.33%)	3 months	-12.66% (-18.37%, -6.32%)	3 months	-1.44% (-10.94%, 7.72%)	3 months	-0.95% (-10.32%, 9.68%)
$PM_{2.5}$	6 months	-0.76% (-11.57%, 9.69%)	6 months	1.14% (-11.76%, 15.02%)	6 months	-2.41% (-13.67%, 9.52%)	6 months	-15.91% (-21.85%, -9.67%)	6 months	0.19% (-9.70%, 9.78%)	6 months	0.39% (-9.54%, 11.33%)
	9 months	-0.66% (-10.89%, 9.24%)	9 months	2.91% (-9.32%, 15.62%)	9 months	-1.42% (-12.09%, 9.69%)	9 months	-18.36% (-23.91%, -12.35%)	9 months	-0.16% (-9.58%, 8.97%)	9 months	-1.52% (-10.93%, 9.05%)
	11 months	-0.40% (-10.44%, 9.33%)	11 months	3.53% (-8.46%, 15.73%)	11 months	-1.33% (-11.81%, 9.50%)	11 months	-20.65% (-26.05%, -14.76%)	11 months	-1.39% (-10.61%, 7.50%)	11 months	-3.74% (-12.89%, 6.62%)

Note: Post 11 months refers to pre-covid lockdown

Table S5. NO_2, NO_X, $PM_{2.5}$ Average absolute causal effect urban background and traffic for ULEZ3 in post one, three, six months for ULEZ3

Pollutants	Urban Background						Urban Traffic					
μg m^{-3}	Central		Inner		Outer		Central		Inner		Outer	
NO_2	1 month	0 (-0.59, 0.62)	1 month	-0.52 (-1.28, 0.29)	1 month	0.84 (-0.58, 2.44)	1 month	0.34 (-0.87, 1.44)	1 month	0.15 (-1.17, 1.39)	1 month	0.41 (-0.6, 1.42)
	3 months	0.02 (-0.56, 0.65)	3 months	-0.53 (-1.26, 0.28)	3 months	0.41 (-1.15, 1.95)	3 months	0.28 (-0.9, 1.42)	3 months	0.18 (-1.15, 1.43)	3 months	0.37 (-0.63, 1.38)
	6 months	-0.27 (-0.87, 0.34)	6 months	-0.77 (-1.53, 0.03)	6 months	0.77 (-0.85, 2.24)	6 months	0.79 (-0.39, 1.93)	6 months	0.09 (-1.24, 1.33)	6 months	0.13 (-0.88, 1.14)
NO_X	1 month	-0.35 (-1.57, 0.86)	1 month	-0.77 (-2.24, 0.55)	1 month	2.96 (-3.26, 9.63)	1 month	0.19 (-3.3, 3.66)	1 month	-1.67 (-6.09, 2.8)	1 month	-0.25 (-3.43, 2.97)
	3 months	0.03 (-1.2, 1.24)	3 months	0.08 (-1.36, 1.42)	3 months	0.13 (-6.57, 6.26)	3 months	-0.23 (-3.67, 3.28)	3 months	-1.32 (-5.78, 3.04)	3 months	-0.05 (-3.26, 3.16)
	6 months	-0.54 (-1.78, 0.65)	6 months	-0.63 (-2.07, 0.74)	6 months	0.77 (-5.93, 6.95)	6 months	1.48 (-1.98, 4.99)	6 months	0.18 (-4.29, 4.54)	6 months	0.19 (-3, 3.41)
$PM_{2.5}$	1 month	0.1 (-0.42, 0.62)	1 month	-0.1 (-0.88, 0.68)	1 month	-0.4 (-1.86, 1.19)	1 month	-0.08 (-0.52, 0.38)	1 month	-0.35 (-0.93, 0.22)	1 month	-0.07 (-0.79, 0.67)
	3 months	0.08 (-0.42, 0.61)	3 months	-0.25 (-1.05, 0.52)	3 months	-1.16 (-2.64, 0.41)	3 months	0.03 (-0.41, 0.49)	3 months	-0.22 (-0.8, 0.35)	3 months	-0.65 (-1.38, 0.08)
	6 months	-0.05 (-0.55, 0.48)	6 months	-0.23 (-1.01, 0.56)	6 months	-1.5 (-2.98, 0.07)	6 months	0.2 (-0.25, 0.65)	6 months	-0.11 (-0.69, 0.46)	6 months	-0.71 (-1.43, 0.02)

Table S6. NO_2, NO_X, $PM_{2.5}$ Average relative causal effect urban background and traffic for ULEZ3 in post one, three, six months for ULEZ3

Pollutants	Urban Background						Urban Traffic					
μg m^{-3}	Central		Inner		Outer		Central		Inner		Outer	
NO_2	1 month	0.01% (-2.53%, 2.65%)	1 month	-2.62% (-6.42%, 1.44%)	1 month	3.79% (-2.62%, 11.00%)	1 month	1.00% (-2.56%, 4.25%)	1 month	0.51% (-4.04%, 4.81%)	1 month	1.60% (-2.32%, 5.52%)
	3 months	0.10% (-2.26%, 2.61%)	3 months	-2.45% (-5.86%, 1.33%)	3 months	1.81% (-5.11%, 8.68%)	3 months	0.78% (-2.48%, 3.92%)	3 months	0.60% (-3.73%, 4.64%)	3 months	1.34% (-2.27%, 4.98%)
	6 months	-1.16% (-3.70%, 1.45%)	6 months	-3.80% (-7.53%, 0.15%)	6 months	3.51% (-3.89%, 10.28%)	6 months	2.23% (-1.10%, 5.42%)	6 months	0.29% (-4.14%, 4.42%)	6 months	0.49% (-3.25%, 4.21%)
NO_X	1 month	-1.14% (-5.17%, 2.83%)	1 month	-2.85% (-8.33%, 2.03%)	1 month	8.16% (-8.98%, 26.55%)	1 month	0.31% (-5.24%, 5.82%)	1 month	-2.92% (-10.63%, 4.88%)	1 month	-0.49% (-6.69%, 5.79%)
	3 months	0.09% (-3.68%, 3.79%)	3 months	0.26% (-4.64%, 4.84%)	3 months	0.34% (-17.21%, 16.40%)	3 months	-0.33% (-5.30%, 4.74%)	3 months	-2.16% (-9.48%, 4.99%)	3 months	-0.09% (-5.82%, 5.63%)
	6 months	-1.79% (-5.84%, 2.14%)	6 months	-2.33% (-7.70%, 2.75%)	6 months	2.15% (-16.49%, 19.33%)	6 months	2.25% (-3.01%, 7.59%)	6 months	0.32% (-7.59%, 8.03%)	6 months	0.37% (-5.86%, 6.66%)
$PM_{2.5}$	1 month	1.33% (-5.45%, 8.10%)	1 month	-1.23% (-11.05%, 8.55%)	1 month	-4.72% (-22.18%, 14.22%)	1 month	-0.84% (-5.65%, 4.18%)	1 month	-4.05% (-10.59%, 2.55%)	1 month	-0.79% (-9.45%, 8.00%)
	3 months	1.01% (-5.12%, 7.42%)	3 months	-2.88% (-12.17%, 6.02%)	3 months	-13.14% (-29.97%, 4.71%)	3 months	0.30% (-4.29%, 5.11%)	3 months	-2.45% (-8.70%, 3.86%)	3 months	-7.47% (-15.72%, 0.91%)
	6 months	-0.64% (-6.46%, 5.54%)	6 months	-2.53% (-11.21%, 6.21%)	6 months	-16.37% (-32.51%, 0.78%)	6 months	1.94% (-2.46%, 6.49%)	6 months	-1.20% (-7.25%, 4.82%)	6 months	-7.77% (-15.74%, 0.19%)

Table S7. Method comparisons for NO_2 for ULEZ1

NO_2 (μg/m^{-3}) in ULEZ1	Policy effect		Policy effect from ML+ ASCM*	
	Effect on central London	Spillover effect	Effect on central London	Spillover effect
Mayor of London Report (2023)	-34% (~ -28 (μg/m^{-3})	-14% (~ -6 (μg/m^{-3})	-17.0% (~ -11.4 (μg/m^{-3})	-3.3% (~ -1.4 (μg/m^{-3})
Ma et al. (2021)	-1.6% (-5.1%, 0.1%) (Traffic sites)	-2.6% (-3.9%, -1.4%) (Traffic sites)	-17.4% (~ -12.1 (μg/m^{-3})	-3.0% (~ -1.3 (μg/m^{-3})
Prieto-Rodriguez et al. (2022)	-19% (~ -14.9 (μg m^{-3}) (Traffic sites)	-15.5% (~ -5.95 (μg m^{-3}) (Traffic sites)	-17.3% (~ -12.1 (μg/m^{-3})	-3.2% (~ -1.4 (μg/m^{-3})

*Note: The analysis period for the ML+ASCM method is consistent with that used in the referenced literature.

Table S8. Average Number and Proportion of Unique Compliant Vehicles in CC hours Different Years within ULEZ1 Area

Date	Number of vehicles driving in the zone		Proportions of vehicles driving in the zone	
	Non-compliant vehicles	Compliant vehicles	Non-compliant vehicles	Compliant vehicles
Mar-19	35,578	55,457	39.1%	60.9%
Apr-19	26,195	63,185	29.3%	70.7%
May-19	25,610	63,186	28.8%	71.2%
June-19	24,549	62,564	28.2%	71.8%
July-19	23,054	60,844	27.5%	72.5%
Aug-19	21,133	58,994	26.4%	73.6%
Sep-19	22,133	63,721	25.8%	74.2%
Oct-19	21,239	61,537	25.7%	74.3%
Nov-19	21,222	63,575	25.0%	75.0%
Dec-19	20,533	63,499	24.4%	75.6%
Jan-20	18,182	60,572	23.1%	76.9%

Note: Data from the Greater London Authority, Congestion Charge (CC) hours (7:00-18:00, Monday to Friday)

Table S9. Average Number and Proportion of Unique Compliant Vehicles in 24 hours Different Years within ULEZ2 Area

Date	Number of vehicles driving in the zone		Proportions of vehicles driving in the zone	
	Non-compliant vehicles	Compliant vehicles	Non-compliant vehicles	Compliant vehicles
Oct-21	124,000	826,000	13.1%	86.9%
Nov-21	75,000	866,000	8.0%	92.0%
Dec-21	66,000	820,000	7.4%	92.6%
Jan-22	57,000	806,000	6.6%	93.4%
Feb-22	61,000	848,000	6.7%	93.3%
Mar-22	61,000	869,000	6.6%	93.4%
Apr-22	57,000	837,000	6.4%	93.6%
May-22	57,000	871,000	6.2%	93.8%
Jun-22	56,000	868,000	6.1%	93.9%
Jul-22	54,000	842,000	6.0%	94.0%
Aug-22	50,000	794,000	5.9%	94.1%
Sep-22	50,000	850,000	5.6%	94.4%
Oct-22	50,000	852,000	5.6%	94.4%

Note: Data from the Greater London Authority

Table S10. Average Number and Proportion of Unique Compliant Vehicles in 24 hours Different Years within ULEZ3 Area

Date	Number of vehicles driving in the zone		Proportions of vehicles driving in the zone	
	Non-compliant vehicles	Compliant vehicles	Non-compliant vehicles	Compliant vehicles
Jul-23	160,000	1,821,000	8.1%	91.9%
Aug-23	139,000	1,750,000	7.4%	92.6%
Sep-23	93,000	1,881,000	4.7%	95.3%
Oct-23	89,000	1,885,000	4.5%	95.5%
Nov-23	88,000	1,971,000	4.3%	95.7%
Dec-23	83,000	1,913,000	4.2%	95.8%
Jan-24	77,000	1,940,000	3.8%	96.2%
Feb-24	80,000	2,018,000	3.8%	96.2%

Note: Data from the Greater London Authority

Reference

1. TFL, Hourly traffic count data for Marylebone Road during 2017-2023, Avaiable from: https://tfl.gov.uk/corporate/transparency/freedom-of-information/foi-request-detail?referenceId=FOI-1318-2324
